# Nanofiber‐based glaucoma drainage implant improves surgical outcomes by modulating fibroblast behavior

**DOI:** 10.1002/btm2.10487

**Published:** 2023-01-18

**Authors:** Aditya Josyula, Ann Mozzer, Julia Szeto, Youlim Ha, Nicole Richmond, Seung Woo Chung, Sri Vishnu Kiran Rompicharla, Janani Narayan, Samiksha Ramesh, Justin Hanes, Laura Ensign, Kunal Parikh, Ian Pitha

**Affiliations:** ^1^ Center for Nanomedicine Johns Hopkins University School of Medicine Baltimore Maryland USA; ^2^ Department of Chemical and Biomolecular Engineering Johns Hopkins University Baltimore Maryland USA; ^3^ Department of Ophthalmology, Wilmer Eye Institute Johns Hopkins University School of Medicine Baltimore Maryland USA; ^4^ Department of Biology Johns Hopkins University Baltimore Maryland USA; ^5^ Department of Biomedical Engineering Johns Hopkins University School of Medicine Baltimore Maryland USA; ^6^ Departments of Pharmacology and Molecular Sciences, Environmental Health Sciences, Oncology, and Neurosurgery Johns Hopkins University School of Medicine Baltimore Maryland USA; ^7^ Departments of Pharmacology and Molecular Sciences, Infectious Diseases, Oncology, and Gynecology and Obstetrics Johns Hopkins University School of Medicine Baltimore Maryland USA; ^8^ Center for Bioengineering Innovation & Design Johns Hopkins University Baltimore Maryland USA; ^9^ Glaucoma Center of Excellence, Wilmer Eye Institute Johns Hopkins University School of Medicine Baltimore Maryland USA

**Keywords:** fibrosis, glaucoma shunts, nanofibers, ocular biomaterials

## Abstract

Biomaterials are implanted in millions of individuals worldwide each year. Both naturally derived and synthetic biomaterials induce a foreign body reaction that often culminates in fibrotic encapsulation and reduced functional lifespan. In ophthalmology, glaucoma drainage implants (GDIs) are implanted in the eye to reduce intraocular pressure (IOP) in order to prevent glaucoma progression and vision loss. Despite recent efforts towards miniaturization and surface chemistry modification, clinically available GDIs are susceptible to high rates of fibrosis and surgical failure. Here, we describe the development of synthetic, nanofiber‐based GDIs with partially degradable inner cores. We evaluated GDIs with nanofiber or smooth surfaces to investigate the effect of surface topography on implant performance. We observed in vitro that nanofiber surfaces supported fibroblast integration and quiescence, even in the presence of pro‐fibrotic signals, compared to smooth surfaces. In rabbit eyes, GDIs with a nanofiber architecture were biocompatible, prevented hypotony, and provided a volumetric aqueous outflow comparable to commercially available GDIs, though with significantly reduced fibrotic encapsulation and expression of key fibrotic markers in the surrounding tissue. We propose that the physical cues provided by the surface of the nanofiber‐based GDIs mimic healthy extracellular matrix structure, mitigating fibroblast activation and potentially extending functional GDI lifespan.

## 1 INTRODUCTION

Over 13 million patients receive indwelling biomaterial implants annually in the United States.^1^ Advances in biomaterial design, manufacturing, and surface chemistry have vastly improved the safety and range of function of implantable biomaterials.^1^, ^2^ However, despite these advances, biomaterials are susceptible to high failure rates, often due to post‐operative fibrosis. Fibrotic encapsulation by activated fibroblasts is the result of an immunological response to the foreign material, hindering implant and tissue function. Addressing the issue of fibrosis and implant failure is critical, as the demand for implantable biomaterials increases with the aging global population.^3^

Pharmacological targeting of biomaterial‐associated fibrotic events is particularly challenging due to the presence of physiological transport barriers and natural drug clearance mechanisms, often leading to transient gains in the functional lifespan of biomaterials. Fibroblasts, the effector cells in fibrosis, transdifferentiate into myofibroblasts upon receiving activating stimuli and deposit extracellular matrix (ECM), which encapsulates biomaterials. Physical stimuli arising from healthy ECM, such as mechanical compliance and topography, maintain the quiescence phenotype of fibroblasts, whereas mechanical tension and rigidity activate fibroblasts.^4^ The response of fibroblasts to physical cues motivates the development of material‐centric approaches to mitigate fibrotic encapsulation of biomaterials.

Glaucoma is a leading cause of irreversible blindness. The only clinically proven approach to prevent glaucomatous vision loss is to reduce intraocular pressure (IOP).^5^, ^6^ Glaucoma drainage implants (GDIs), which can be classified into those with and without a reservoir for aqueous drainage, are among the most widely used ocular biomaterials.^7^ However, implantation of GDIs for IOP reduction often leads to post‐operative complications, including hypotony and fibrosis.^8^, ^9^, ^10^, ^11^, ^12^ Conventional subconjunctival GDIs have a modest median functional lifespan of 5 years due to fibrosis, and revision or repeat procedures are burdensome for patients and have an increased risk of failure.^13^, ^14^, ^15^ Additionally, the aqueous humor draining into the subconjunctival space contains soluble factors implicated in fibroblast recruitment and activation, such as transforming growth factor‐beta (TGF‐β), platelet‐derived growth factor (PDGF) and vascular endothelial growth factor (VEGF).^16^, ^17^ Furthermore, continuous aqueous humor outflow causes chronic mechanical tension in the bleb, triggering tissue resident fibroblast activation.^18^ The introduction of the implant material only further exacerbates these cellular processes, leading to recognition of the material as foreign, and subsequent fibrotic encapsulation. Indeed, activated fibroblasts were found in fibrotic tissue surrounding glaucoma implants in preclinical surgical models and in glaucoma patients who have a history of implant failure.^19^, ^20^

**FIGURE 1:**
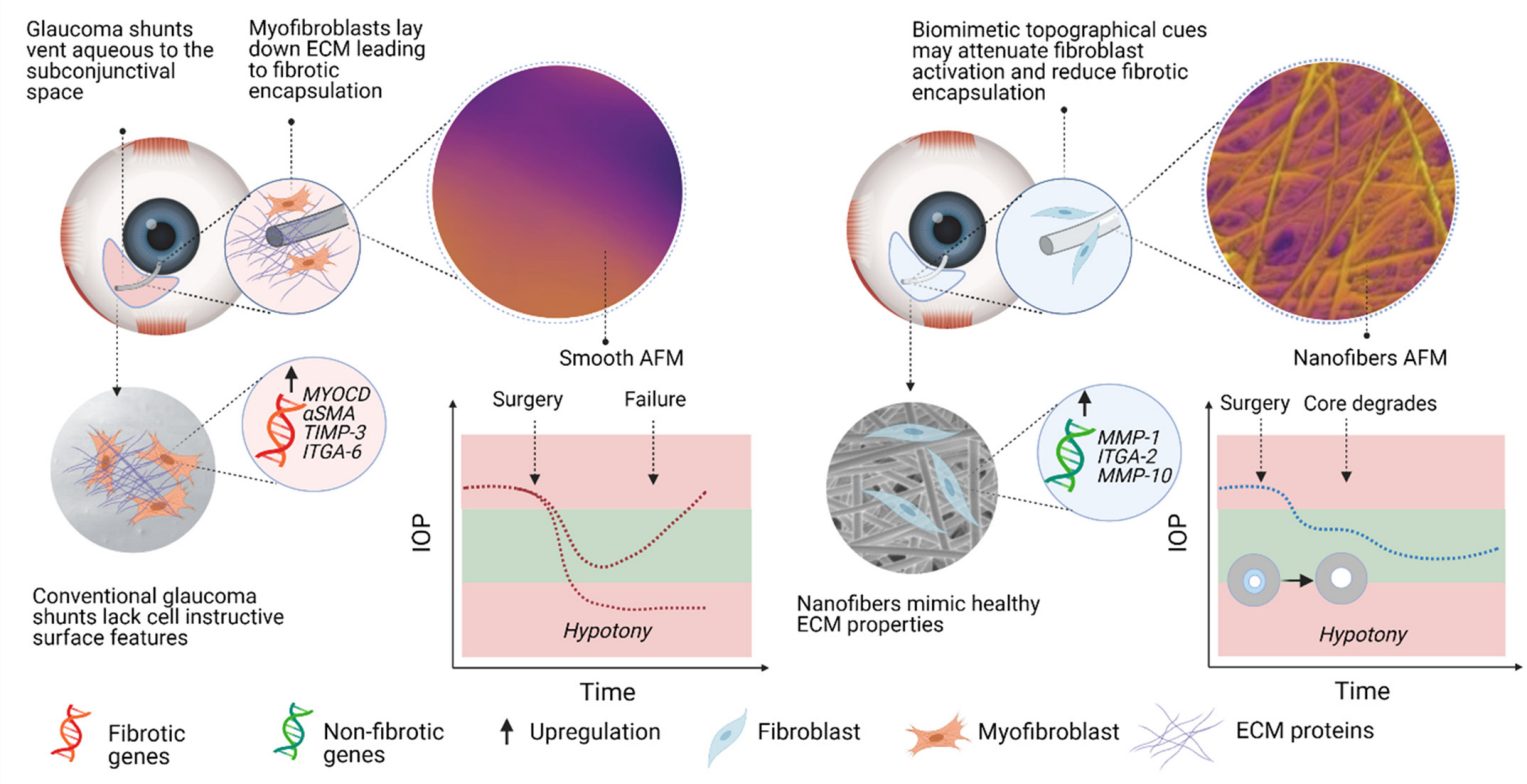
Nanofiber‐based, partially degradable glaucoma drainage implant. Post‐operative hypotony and fibrotic encapsulation lead to complications and failure of glaucoma surgery. Here, we engineered partially degradable, nanofiber‐based GDIs and compared functional outcomes to implants with a smooth surface. We aimed to prevent hypotony and limit aqueous outflow in the acute post‐operative phase using a degradable core design. We further hypothesized that imparting nanofiber architecture to glaucoma GDIs to mimic healthy ECM would support fibroblast quiescence to preclude the fibrotic processes that lead to device failure.

Thus, there is significant need to engineer GDIs which enable (1) safe and effective IOP lowering, (2) controlled aqueous outflow to avoid hypotony and reduce subconjunctival exposure to aqueous cytokines, and (3) mitigation of fibrosis (Figure 1). Here, we utilize a versatile nanofiber‐based platform to manufacture tube GDIs. We hypothesized that a surface composed of nanofibers would be more structurally and mechanically similar to healthy ECM, and thus, less likely than smooth surfaces to be perceived by fibroblasts as foreign. In cell culture and in rabbit eyes, the nanofiber architecture reduced fibroblast activation and increased cellular integration. Additionally, we demonstrate that tube GDIs with a nanofiber surface reduced subconjunctival fibrosis in the eyes of rabbits compared to GDIs with smooth surfaces. We then evaluated the performance of the nanofiber‐coated GDIs in comparison to the clinically available, minimally invasive XEN® Gel Stent, and the silicone tube portion of the widely used Baerveldt® GDI (BGI tube). We observed that nanofiber‐based GDIs were capable of safe and effective IOP reduction, while minimizing conjunctival fibrosis‐related gene expression. We anticipate that the reduced fibrotic response to nanofiber‐based tube GDIs could lead to improved functional lifespan and positive long‐term outcomes in glaucoma surgery.

## 2 RESULTS

### 2.1 Nanofiber‐based stents retain architecture and promote cell integration in vivo

**FIGURE 2:**
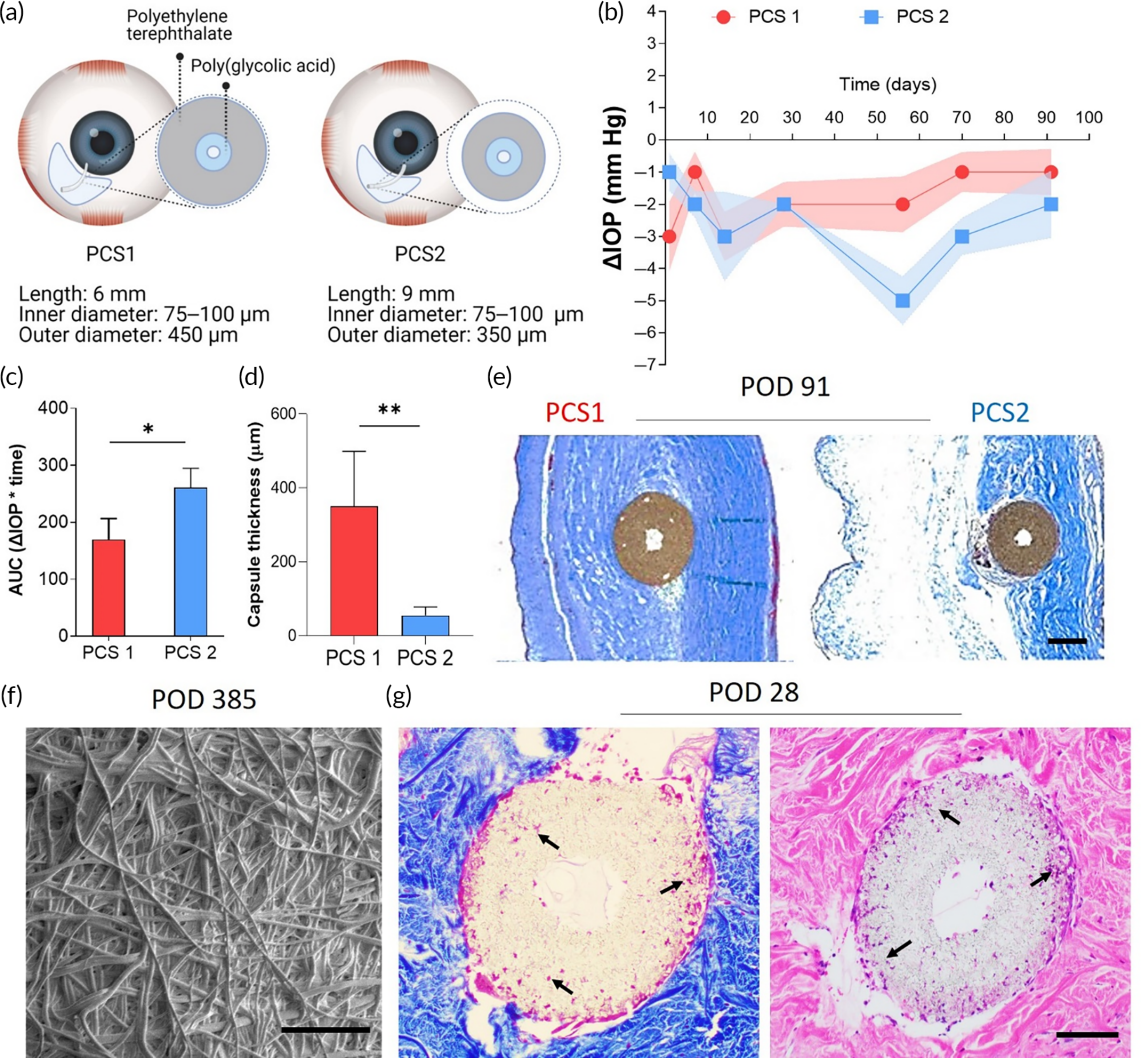
Nanofiber‐based GDIs achieve effective IOP lowering while avoiding hypotony and reducing subconjunctival fibrosis. (a) Schematic view of nano‐structured pressure control shunts (PCS) with a degradable PGA nanofiber core designed to prevent hypotony. (b) IOP lowering profiles of PCS1 and PCS2 in normotensive NZW rabbits. (c) Cumulative IOP reduction (area under the curve [AUC]) and (d) collagen capsule thickness of PCS1 and PCS2 91 days after GDI placement. (e) Masson's trichrome staining illustrating subconjunctival collagen deposition around PCS1 and PCS2 at post‐operative day 91. Scale bar represents 200 μm. (f) SEM analysis of explanted PCS2 GDIs at post‐operative day 385 showed that PET nanofiber architecture was preserved without apparent protein deposition. Scale bar represents 100 μm. (g) Masson's trichrome (left) and H&E (right) staining of PCS GDIs at post‐operative day 28 showed cells integrating within the walls of the nanofiber‐based GDI. Scale bar represents 100 μm.

We previously reported the development of nanofiber‐based glaucoma GDIs with a degradable PGA core to enable controlled outflow of aqueous humor.^21^ These pressure control shunts (PCS) prevent acute post‐operative hypotony and allow for greater volumetric outflow at later post‐operative stages for sustained IOP reduction.^21^ In order to evaluate the effect of device stiffness and dimensions on GDI efficacy, we developed and evaluated two PET/PGA nanofiber‐based designs in a long‐term rabbit model: (1) a shorter GDI with a thicker, more rigid wall (PCS1: length 6 mm, OD 450 μm) and (2) a longer, more flexible GDI (PCS2: length 9 mm, OD 350 μm) (Figure 2a). PCS2 showed a greater maximal ΔIOP of −5 ± 1.3 mmHg (~30% lower than the non‐operated contralateral eye at Day 56) compared to −3 ± 1.7 mmHg for PCS1 (Figure 2b). Similarly, PCS2 provided ~1.5‐fold increased cumulative ΔIOP (area under the curve, AUC) compared to PCS1 over the 91‐day study period (Figure 2c). Histological analysis of conjunctival tissue on post‐operative day (POD) 91 revealed a capsule thickness of 55 ± 23 μm in the PCS2 group compared to 350 ± 149 μm in the PCS1 group (Figure 2d), where the reduced capsule thickness was associated with an edematous subconjunctival space (Figure 2e). SEM analysis of GDIs explanted on POD 385 revealed that the PET nanofiber architecture was preserved without discernible protein deposition or implant encapsulation (Figure 2f). In separate studies, histological analysis of scleral tissue surrounding the GDIs at POD 28 showed cells integrated within the surface nanofiber layers (Figure 2g). The observations of cell integration and conservation of the nanofiber surface topography after more than 1 year of implantation were of particular interest, given that even commercially used GDIs have been shown to fail due to fibrotic encapsulation after only 1–2 months in rabbits.^22^, ^23^, ^24^

### 2.2 Characterization of fibroblast interactions with nanofiber and smooth scaffolds in vitro

**FIGURE 3:**
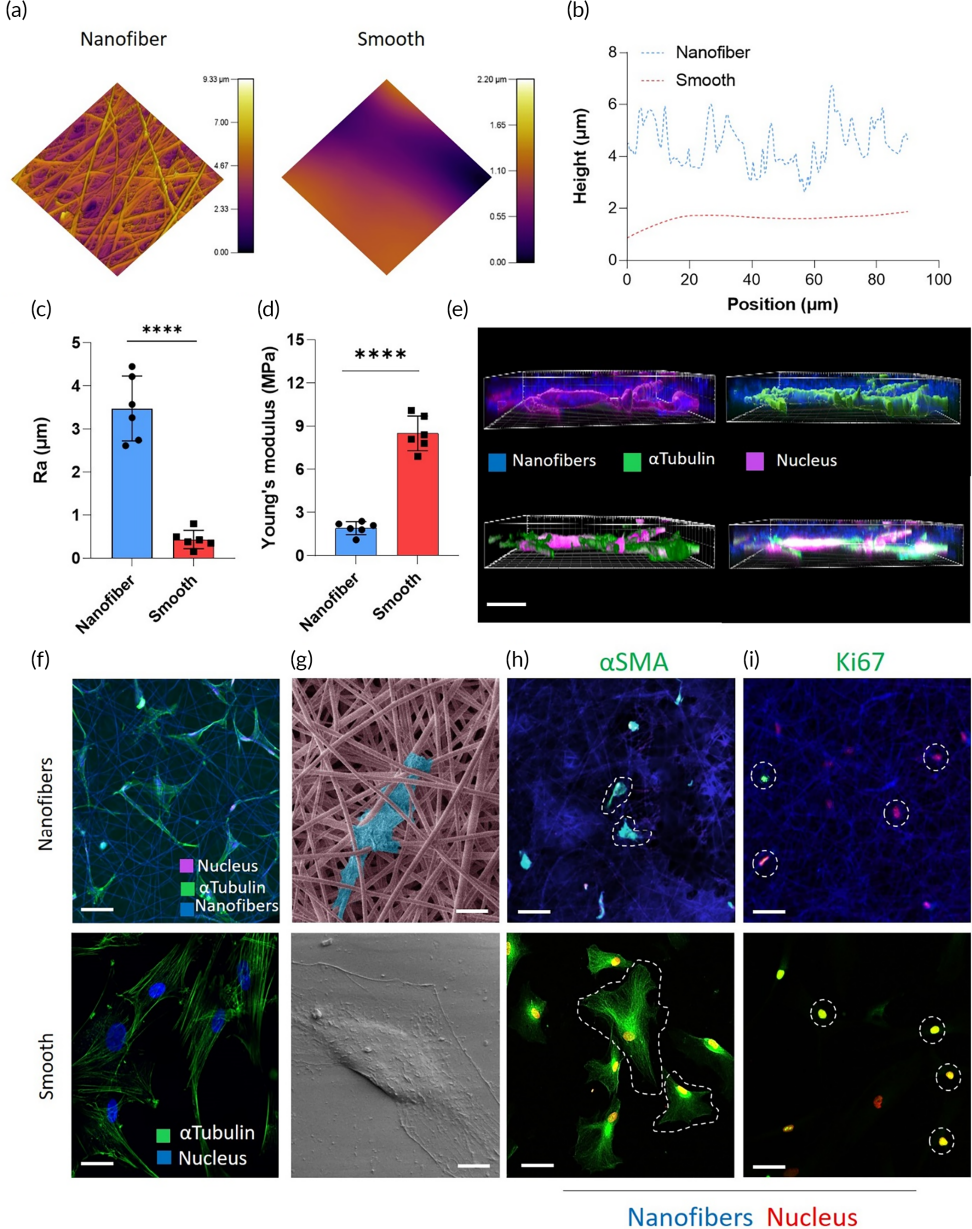
Nanofiber scaffolds promote scleral fibroblast integration and attenuate activation. (a) AFM scans of the surface topography of nanofiber and smooth surfaces and (b) height profiles indicating surface roughness of nanofiber and smooth surfaces. (c) Quantification of the dimensional roughness parameter (Ra). ****p ≤ 0.0001, student's *t* test. (d) Young's modulus of nanofiber and smooth scaffolds. ****p ≤ 0.0001, student's *t* test. (e) 3D reconstructed images from z‐stacks depicting the volume distribution of fibroblast actin and nuclei in nanofiber scaffolds. Fibroblasts were interspersed between nanofibers and penetrated through the depth of nanofiber scaffolds. Scale bars represent 150 μm. Cells morphology and interaction with nanofiber and smooth scaffolds were visualized using (f) confocal microscopy (scale bars represent 50 μm) and (g) SEM (fibroblast false colored blue, nanofibers false colored pink). Scale bar represents 50 μm. TGF‐β stimulated scleral fibroblasts were cultured on either nanofiber or smooth scaffolds and stained for (h) αSMA and (i) Ki67. Yellow represents overlap of green staining for αSMA and Ki67 with the red nuclei. Scale bars represent 50 μm.

The results of PCS2 evaluation led us to hypothesize that designing flexible GDIs with nanofiber surfaces may improve the functional device lifespan. However, the fully nanofiber‐based PCS GDIs had mechanical limitations observed by the surgeon during handling and implantation, including irreversible deformation under compressive stress. Further, PET was too mechanically rigid when heated to evaluate a smooth‐surfaced GDI in vivo (not shown). Thus, we incorporated polyurethane (PU) in later designs, due to its increased mechanical resiliency and flexibility when annealed into smooth surfaces. Further, smooth PU surfaces were amenable to coating with electrospun nanofibers post‐annealing, which allowed us to directly compare smooth and nanofiber surface architecture. To characterize the effect of surface topography on cell activation in vitro, we electrospun PET and PU nanofiber scaffolds (Figure S1a) that were annealed to either maintain the nanofiber network under hydration or heated to the melting point and gradually cooled to room temperature to create smooth surfaces (Figure S1b). Nanofiber diameters, analyzed using SEM micrographs, ranged from 400 to 1000 nm (Figure S1c). AFM images (Figure 3a) and height mapping (Figure 3b) of the PU nanofiber scaffolds showed an arithmetic average surface roughness (Ra) of 3.75 ± 0.75 μm, whereas the smooth scaffolds had an Ra of 0.43 ± 0.21 μm (Figure 3c). Mechanical testing of the PU scaffolds showed that the nanofibers displayed a significantly lower Young's modulus (1.9 ± 0.4 MPa) as compared to smooth scaffolds (8.5 ± 1.2 MPa) (Figure 3d, *p* < 0.0001). A similar trend was found for nanofiber and smooth PET scaffolds (Figure S1d). Protein adsorption was significantly lower on both PU and PET nanofiber scaffolds compared to their smooth counterparts (Figure S1e). To study the effect of nanoscale features on ocular fibroblasts, primary human scleral fibroblasts were cultured on nanofiber and smooth scaffolds. False volume images of 3D reconstructed z‐stacks showed fluorescent staining for α‐tubulin (green) and cell nuclei (purple) throughout the ~150 μm nanofiber (blue) scaffold thickness (Figure 3e). Fibroblasts imaged using confocal microscopy (Figure 3f and Figure S2a) and SEM (Figure 3g and Figure S2b) showed fibroblasts integrated within the nanofiber matrix and displaying multi‐directional points of adhesions with multiple large lamellipodia, while fibroblasts on smooth surfaces had a flat, spindle‐shaped appearance. Further, fibroblasts on nanofibers had a more branched appearance with significantly smaller cell bodies (area 1004 ± 431 μm^2^ [Figure S2c], mean ferret diameter 154 ± 28 μm [Figure S2d]) and a more branched appearance in comparison to cells on smooth scaffolds (area 4302 ± 922 μm^2^ [Figure S2c], mean ferret diameter 36 ± 12 μm [Figure S2d]). In order to ascertain how these interactions affect cell behavior, TGF‐β activated scleral fibroblasts were stained for the activation marker αSMA (Figure 3h), as well as the proliferation marker Ki67 (Figure 3i). Qualitatively, nanofibers attenuated activation and proliferation of scleral fibroblasts in vitro compared to smooth surfaces.

### 2.3 Characterization of fibroblast transcriptional response in vitro

**FIGURE 4:**
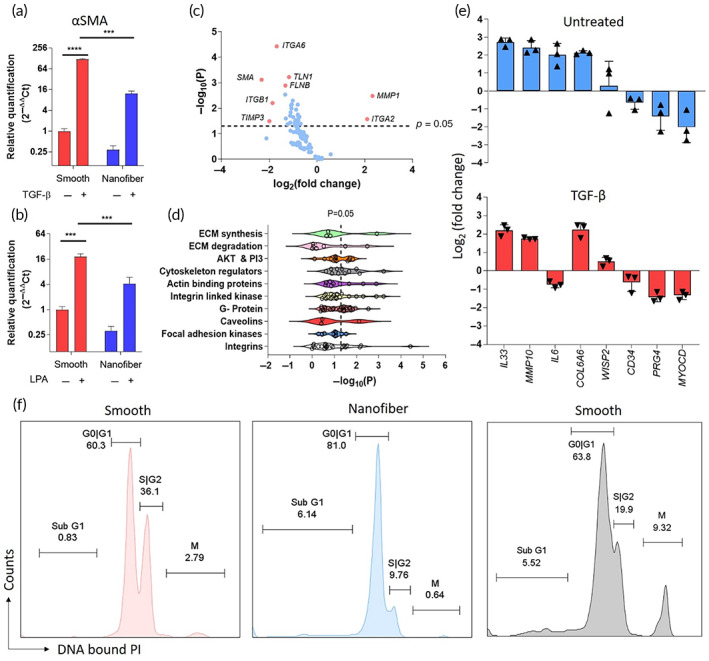
Nanofibers promote quiescence in scleral fibroblasts. Nanofibers significantly attenuated αSMA expression in fibroblasts in response to activation with either (a) TGF‐β or (b) LPA in comparison to fibroblasts cultured on smooth scaffolds. (c) Volcano plot of relative expression of genes associated with focal adhesion pathways for fibroblasts cultured on nanofibers relative to smooth scaffolds. *p ≤ 0.05, ***p ≤ 0.001, ****p ≤ 0.0001 by ANOVA for data in (a), (b), and (c). (d) Gene ontology assignment of genes differentially expressed by cells cultured on nanofibers relative to smooth scaffolds. (e) Relative expression of key genes previously associated with positive surgical outcomes by fibroblasts cultured on nanofibers compared to smooth scaffolds. (f) Propidium iodide‐based flow cytometry analysis of the cell cycle showed that nanofibers induce reversible G1 arrest. Fibroblasts cultured on a smooth surface (left panel) were transferred to a nanofiber scaffold (middle panel) for 24 h followed by a return to a smooth surface (right panel) for an additional 24 h. Inset numbers indicate percentage of total fibroblasts present in respective phases of cell cycle.

We next sought to characterize the fibrosis‐related transcriptomic changes underlying the differences in cell morphology observed in response to nanofiber and smooth scaffolds. Fibroblasts cultured on nanofibers displayed a 4‐fold lower expression of αSMA in the absence of stimulation (Figure 4a, *p* < 0.001) as compared to smooth surfaces. Stimulation with the fibroblast activators TGF‐β or LPA for 24 h resulted in robust upregulation of αSMA in fibroblasts cultured on smooth surfaces, while the increase in αSMA expression was significantly attenuated on both PET (Figure 4b) and PU (Figure S2e) nanofiber scaffolds. qRT‐PCR was then used to measure mRNA transcript levels associated with focal adhesion signaling and ECM synthesis. Cells cultured on nanofibers displayed a 4.2‐fold increase in *ITGA2* (*p* = 0.031) and a 5‐fold increase in *MMP‐1* expression (*p* = 0.003) (Figure 4c). It is notable that as a collagen‐binding integrin, *ITGA2* is also an important regulator of integrin signaling, and *ITGA2* knockout mice display an enhanced fibrotic response.^25^ Additionally, MMP1 is a protein known to degrade ECM.^26^ We also observed decreased expression of the matrix stiffness sensor *ITGA6* (3.2‐fold; *p* < 0.0001) and profibrotic marker *ITGB1* (3.64‐fold; *p* = 0.006) in fibroblasts cultured on nanofibers as compared to cells cultured on smooth surfaces (Figure 4c). These data, along with differential expression of transcripts associated with downstream actin signaling indicate that nanofibers potentially act through central integrin‐mediated sensing pathways to instruct cell behavior. Furthermore, significant differential expression in pathways including ECM synthesis and degradation, actin, and integrin‐mediated signaling was observed in cells cultured on nanofiber scaffolds as compared to smooth surfaces (Figure 4d). A histogram showing differential expression of 100 genes associated with focal adhesion signaling and fibrosis in fibroblasts cultured on nanofibers is shown in Figure S3a. We then profiled the fibroblast response to the scaffolds using a cassette of genes identified previously as a signature of fibrotic glaucoma surgeries.^27^ In the absence of TGF‐β (untreated), fibroblasts cultured on nanofibers had increased levels of *IL‐33*, *MMP‐10*, *IL‐6*, and *COL6A6* transcripts (Figure 4e) which have been associated with successful, non‐fibrotic outcomes.^27^ After TGF‐β treatment, this pattern was preserved with the exception of *IL‐6* (Figure 4e). Additionally, nanofibers significantly attenuated the expression of the pro‐fibrotic marker *MYOCD* ^28^, ^29^ under both stimulated and unstimulated conditions (Figure 4e). ANOVA *p*‐values associated with this analysis are shown in Figure S3b.

### 2.4 Effect of nanofibers on fibroblast cell cycle in vitro

The broad transcriptomic changes in fibroblasts in response to the nanofiber and smooth scaffolds suggested globally regulated phenomena. In other cell types, including pluripotent stem cells, tissue‐specific progenitor cells, and adaptive immune cells, resistance to activating stimuli is associated with quiescence.^30^ We hypothesized that physical cues from nanofibers induce quiescence in fibroblasts. To explore this hypothesis, we analyzed the cell cycle of fibroblasts exposed to nanofibers or smooth surfaces using flow cytometry. When fibroblasts were cultured on smooth PET surfaces for 24 h, approximately 60% of the cell population were present in the G0/G1 phase and ~36% of cells in S/G2 phase (Figure 4f) indicating normal cell cycle progression. When transferred to nanofiber scaffolds for a period of 24 h, the percentage of fibroblasts in the G0/G1 phase increased to 81% and the percentage of cells in the S/G2 phase reduced to ~9% (Figure 4f). When the cells were then transferred back to smooth scaffolds, within 24 h the percentage of cells in the G0/G1 phase was then reduced to ~64% (Figure 4f), indicating that nanofibers induced cell cycle arrest. Moreover, after culturing cells for 48 h on the nanofibers, ~31% of cell population exited the cell cycle, indicating induction of a quiescence phenotype (Figure S4). Taken together, these data suggest that exposure of cells to nanofibers promoted a quiescent, non‐fibrotic phenotype.

### 2.5 Nanofiber‐based GDI reduces subconjunctival fibrosis in rabbits

**FIGURE 5:**
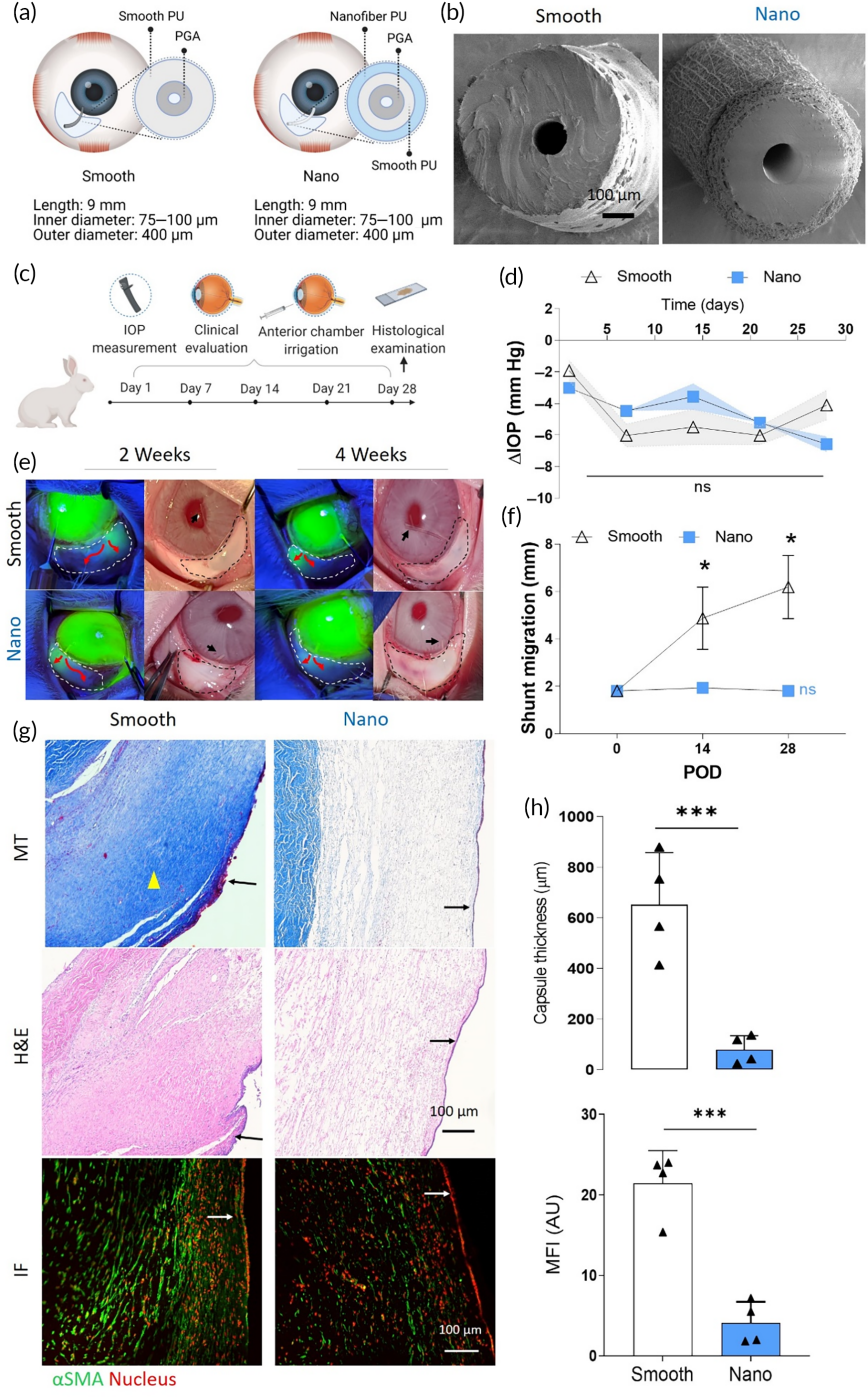
Comparative analysis of the functional outcomes of smooth versus nanofiber GDIs. (a) Schematic view and dimensions of GDIs. (b) SEM images of GDIs with smooth (Smooth) and nanofiber (Nano) exteriors. (c) IOP, patency, migration, and clinical grading of subconjunctival blebs were recorded over a period of 28 days to compare functional efficacy (*n* = 4, each). (d) IOP outcomes of 9 mm long Nano and Smooth GDIs. (e) Left panels: evaluation of GDI patency by anterior chamber irrigation with fluorescein sodium. White dashed lines denote the subconjunctival bleb and red arrows indicate direction of aqueous outflow. Right panels: blebs (dashed lines) formed by aqueous drainage via distal end (black arrow) of implants in the anterior chamber of rabbit eyes at post‐operative days 14 and 28. (f) Measurement of implant migration into the anterior chamber of the eye. *p*‐values were calculated using student's *t* test comparing post‐operative day 28 and day 0 values independently for each group. (g) Representative MT, H&E, and IF images of tissue surrounding Smooth and Nano GDIs at post‐operative day 28. Scale bars represent 100 μm. Yellow arrowheads indicate collagen capsules and arrows indicate conjunctival epithelium. (h) Thickness of collagen capsules surrounding GDIs and quantification of mean fluorescence intensity (MFI) of the fibroblast activation marker αSMA measured at post‐operative day 28. ***p ≤ 0.001 by student's *t* test for data in (e), (f), and (h)

Subconjunctival fibrosis is a key determinant of outcomes in glaucoma surgery. The thickness of scar tissue surrounding GDIs is negatively correlated with the implant's ability to facilitate fluid venting.^31^ Failed implants often have a thick barrier of fibrotic tissue that prevents fluid outflow, whereas functional implants are surrounded by edematous tissue that signifies active fluid outflow. In order to study the effect of nanofibers on subconjunctival fibrosis, we manufactured three similarly designed PU GDIs with dimensions informed by the prior PCS studies and either a smooth (ID 75 → 100 μm, OD 400 μm, length 9 mm) or nanofiber exterior (ID 75 → 100 μm, OD 400 μm, length 9 and 12 mm) (Figure 5a). In addition, to improve upon the mechanical recovery of the tubular structure from compressive forces, the nanofiber‐based GDIs (Nano) were manufactured with an inner layer of PU that was annealed and then coated with nanofibers (Figure 5b). GDIs were implanted in normotensive rabbits to vent aqueous humor from the anterior chamber to a surgically created space under the conjunctiva (bleb) and monitored for a period of 28 days (Figure 5c). No statistically significant differences in IOP lowering profile (Figure 5d and Figure S5a) or cumulative IOP reduction (Figure S5b) were found between the three groups. Anterior chamber irrigation with fluorescein sodium revealed active implant drainage in all groups at 2 and 4 weeks post‐operatively (Figure 5e and Figure S5c). However, in rabbits which received Smooth GDIs (4 out of 4), significant peritubular leakage was observed, which was minimal with both the 9 mm and 12 mm Nano GDIs (1 out of 4) (Figure S5d). This is potentially a significant contributor to IOP reduction in eyes that received smooth GDIs. Additionally, Smooth GDIs migrated ~4 mm into the anterior chamber (*p* < 0.05 as compared to D0 position), while the Nano GDIs did not show appreciable migration (Figure 5f and Figure S5e), suggesting increased tissue integration. Bleb extent and height (Figure S5f) observed at POD 14 was preserved until the end of the study in all groups. Masson's trichrome staining (Figure 5g) of tissue surrounding smooth GDIs revealed abundant collagen deposition with a capsular thickness of 610 ± 161 μm (Figure 5h), whereas the subconjunctival space surrounding the 9 mm Nano GDIs was edematous (Figure 5g) with a capsule thickness of 79 ± 45 μm (*p* = 0.0004) (Figure 5h). Nano GDIs 12 mm in length had greater but nonsignificant collagen deposition in the subconjunctival space compared to the 9 mm Nano GDIs (Figure S5g). Quantitative analysis of mean fluorescence intensity (MFI) from αSMA stained IF images showed that the smooth GDI increased fibroblast activation compared to the Nano GDI (Figure 5h).

### 2.6 Comparative evaluation of nanofiber‐based and commercially available GDIs

**FIGURE 6:**
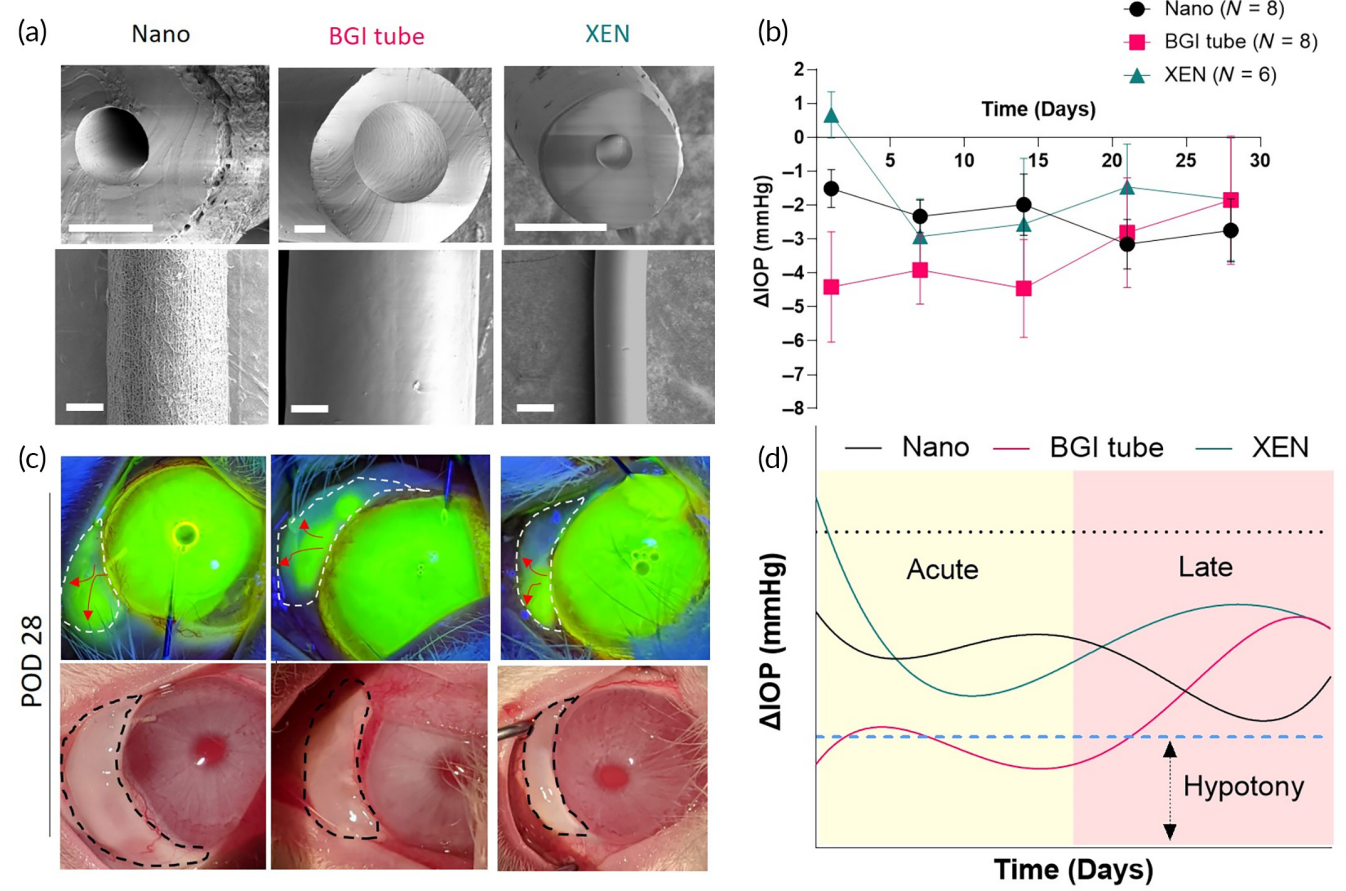
In vivo evaluation of efficacy and patency of nano‐structured GDIs compared to commercially available GDIs. (a) SEM images showing luminal (top) and side (bottom) views of BGI tube, XEN, and Nano GDIs. Scale bars represent 100 μm. (b) Post‐operative IOP change from baseline levels. (c) Active subconjunctival drainage as confirmed by anterior chamber irrigation with fluorescein sodium (top) and representative gross images of subconjunctival blebs (bottom) 28 days after GDI placement. Black arrowheads show distal end of GDIs in the anterior chamber. (d) Best fit (fourth‐order polynomial) curves in acute and late post‐operative periods. Black dashed line indicates baseline IOP and the blue line indicates hypotony.

Next, we sought to evaluate the performance of the leading 9 mm Nano GDI in comparison to clinically available GDIs. We chose two of the most commonly used subconjunctival GDIs: the silicone tube portion of Baerveldt Glaucoma Implant (BGI tube) and the XEN Gel Stent. The BGI tube and the XEN have markedly different IOP lowering profiles,^11^, ^32^, ^33^ composition (silicone and porcine collagen), and dimensions (BGI tube: OD 635 μm, ID 300 μm; XEN: OD 150 μm, ID 40 μm). SEMs of the cross‐sectional and longitudinal views of the devices highlighted the smooth exterior of the BGI tube and XEN GDIs compared to the Nano GDI (Figure 6a). We implanted either the Nano GDI (*n* = 8), the BGI tube (*n* = 8), or the XEN gel stent (*n* = 6) in normotensive rabbit eyes and monitored for 28 days. While no immediate hypotony was observed in the XEN and Nano GDI groups, persistent hypotony was seen in the BGI tube group up to POD 14. Nano GDIs progressively reduced IOP over time (Figure 6b) in a controlled and gradual manner. XEN and BGI tube groups, in contrast, trended toward less IOP reduction over time because of post‐operative fibrosis (Figure 6b). Notably, on POD 1, IOP lowering was not evident in eyes that received the XEN GDI, whereas a mean IOP lowering from baseline of 1.5 ± 0.5 mmHg was observed in the Nano GDI group. No statistically significant difference in IOP lowering was observed between XEN and Nano GDI groups at PODs 7 and 14 (Figure 6b). Beyond POD 14, IOP in the BGI tube group trended toward baseline with significantly greater IOP at POD 28 as compared to POD 1 (Figure 6b). The mean IOP reduction from baseline at POD 28 in the Nano GDI group (−2.7 ± −0.9 mmHg) was lower than the BGI tube (−1.8 ± −1.8 mmHg) and the XEN (−1.8 ± −1.8 mmHg) (Figure 6b). Bleb morphology in the XEN and Nano groups was largely unchanged through POD 28 (Figure 6c and Figure S6a,b). Best fit curves, generated using fourth‐order polynomial equation fitting, highlight the markedly different IOP lowering profile of the Nano GDI as compared to the XEN and BGI tube groups (Figure 6d). The BGI tube group showed drastic and immediate post‐operative IOP reduction, which persisted for 14 days and trended toward baseline pressure in later post‐operative stages. Further, the total AUC of the IOP reduction over 28 days was significantly higher in the Nano group versus the XEN (Figure S6c). No statistically significant difference was observed between Nano and BGI tube groups due to the persistent post‐surgical hypotony observed in the BGI tube group (Figure S6c). Further, peritubular leakage in the BGI tube group gave rise to large blebs with elevation at POD 28 significantly greater, as compared to POD 14 (Figure S6a). Consistent with previous experiments, Nano GDIs displayed significantly lower migration rates in comparison to BGI tube and XEN (Figure S6d). A complete list of adverse events observed with each GDI is shown in Figure S6e.

**FIGURE 7:**
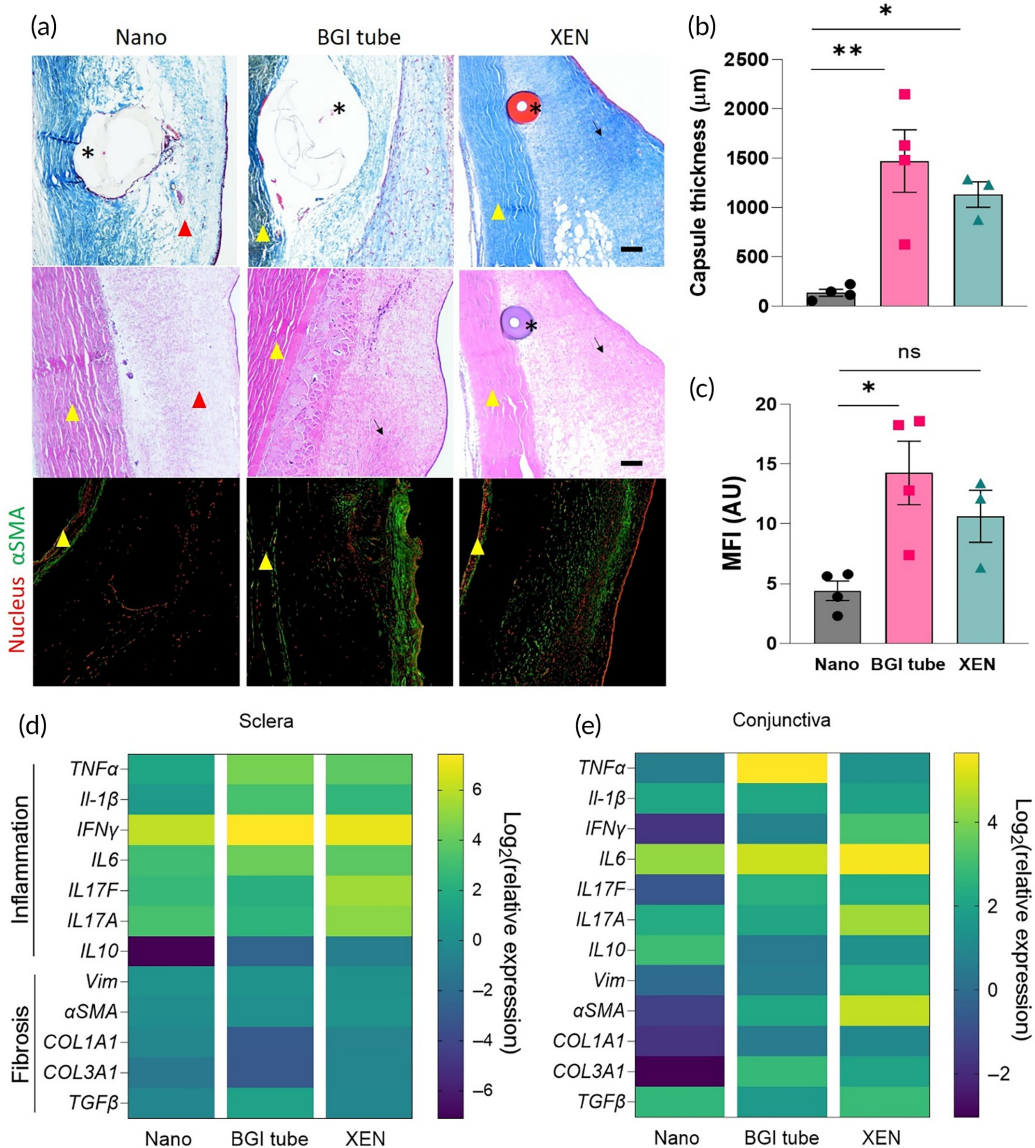
Comparative histology and transcript analysis of Nano versus commercially available GDIs. (a) Representative MT, H&E, and immunofluorescence images of tissue surrounding BGI tube, XEN, and Nano GDIs at post‐operative day 28. Scale bars represent 100 μm. (b) Quantification of collagen capsule thickness and (c) mean fluorescence intensity of αSMA signal in the subconjunctival space surrounding the GDIs. *p ≤ 0.05, **p ≤ 0.01 by ANOVA for data in (b) and (c). qRT‐PCR analysis of putative inflammatory and fibrotic factors in addition to the immunoregulatory IL‐10 measured in the (d) sclera and (e) conjunctiva surrounding the GDIs relative to expression in healthy tissue

Histological analysis using Masson's trichrome staining showed dense collagen capsules surrounding the XEN and BGI tube GDIs in the subconjunctival space on Day 28 (Figure 7a). Further, abundant αSMA positive fibroblast populations were observed surrounding the XEN and BGI tube implants, whereas the subconjunctival space surrounding Nano GDIs was largely edematous and depleted of αSMA positive fibroblasts (Figure 7a). This was corroborated by reduced collagen capsule thickness in the Nano group (136 ± 70 μm) compared to the XEN (1133 ± 223 μm) and BGI tube groups (1472 ± 631 μm) (Figure 7b) and reduced MFI measurements for αSMA in the Nano GDI group (Figure 7c). We further characterized gene expression levels of transcripts associated with inflammation (*TNF‐α*, *IL‐1β*, *IFNγ*, *IL6*, *IL10*) and fibrosis (vimentin [*vim*], collagen types 1 and 3 (*COL1A1*, *COL3A1*), *αSMA*, *TGF‐β*, *IL17A*, and *IL17F*) in tissues collected at Day 28 compared to healthy tissues. Scleral expression (Figure 7d) of transcripts associated with inflammation and fibrosis was largely similar among the groups, as the sclera is not the site of post‐operative fibrosis in glaucoma surgery. In contrast, conjunctival expression of the fibrotic markers *Vim* (55‐fold and 21‐fold), *αSMA* (32‐fold and 8‐fold), *COL1A1* (14‐fold and 15‐fold), and *COL3A1* (14‐fold and 40‐fold), as well as the inflammation marker *IFNγ* (30‐fold and 11‐fold) were significantly downregulated in the Nano GDI group as compared to the XEN and BGI tube groups, respectively (Figure 7e). Notably, mRNA expression of the immunoregulatory cytokine IL‐10 was significantly elevated in the conjunctiva of the Nano GDI group as compared to XEN and BGI tube GDIs (2.5‐fold and 4.8‐fold, respectively) (Figure 7e). *p*‐values comparing expression levels between the three groups can be found in Table S4. These data suggest that the nanofiber surface also attenuated fibroblast activation in response to the GDI in the conjunctival tissue in vivo.

## 3 DISCUSSION

A wide range of biomaterials from bioinert metals to synthetic polymers have been applied to GDIs that have been studied in both preclinical models and clinical trials. Post‐operative outcomes across the range of different types of GDI materials^34^, ^35^, ^36^ highlight the need for alternative material‐centric strategies to prevent biomaterials‐associated fibrosis. Scleral, conjunctival, and tenon's fibroblast responses to fibrotic stimuli have been characterized previously.^4^, ^27^, ^37^, ^38^ Specifically, VEGF production in response to fibrotic stimuli as well as in vitro proliferation responses to various small molecule drugs have been well characterized in tenon's fibroblasts.^39^ Additionally, conjunctival and scleral fibroblast sub‐populations have been shown to be immunomodulatory and involved in tissue remodeling and collagen synthesis.^38^, ^40^, ^41^, ^42^ Notably, scleral fibroblasts produce a robust pro‐fibrotic response to activating stimuli such as TGF‐β.^43^ Fibroblast migration from the scleral and subconjunctival tenon's fascia into the subconjunctival space is a likely source of fibrosis following implantation of trans‐scleral GDIs. The deposition of collagen and other ECM proteins, limits the functional capacity of GDIs.^31^ While there are therapeutics that target fibroblast activation,^4^, ^43^ adjunct use of existing cytotoxic antimetabolite drugs, such as MMC, is transient and can lead to complications.^9^, ^44^, ^45^, ^46^ Additionally, achieving local delivery of anti‐fibrotic agents at sufficient concentrations for the duration of GDI implantation is a challenge. Alternatively, there is significant potential to leverage the cellular response to physical stimuli to prevent biomaterial‐induced fibrosis. The ECM interacts with cells through ligand‐based signaling as well as structural, topographical, and mechanical cues. Rather than continuous generation of chemical signals from cells, these physical cues can instruct migrating cells to phenocopy tissue resident quiescent cells to control proliferation and stop downstream signaling. Exploiting these mechanisms to develop biomaterials that mimic healthy ECM properties may mitigate fibrotic events by supporting quiescence.^47^, ^48^, ^49^, ^50^, ^51^ For example, incorporation of nano‐scale texture into breast implants showed reduced sub‐muscular capsular contracture in clinical studies.^51^, ^52^ ECM in healthy tissues is composed of fibrils that range from 10 nm to 1 μm in width with pore sizes between 1 and 2 μm.^53^ Using electrospun nanofibers (400–1000 nm diameter), we constructed scaffolds and GDIs with topographical properties similar to that of healthy ECM, which skewed fibroblasts to a non‐fibrotic phenotype in vitro even in the presence of activating stimuli, and in rabbit eyes. Similarly, our results demonstrate that GDIs mimicking the topography and other properties of healthy ECM significantly reduce fibroblast proliferation in vitro and encapsulation in vivo. Thus, structural modification, rather than pharmacological intervention, may be a more promising approach to extending the functional lifespan of GDIs.

TGF‐β is a potent fibroblast activator that is implicated in fibrosis following placement of conventional GDIs.^34^, ^35^, ^54^, ^55^ By binding to cell surface receptors on fibroblasts, TGF‐β triggers transdifferentiation of dormant fibroblasts into myofibroblasts, which are characterized by increased cell contractility and size, ECM remodeling, and expression of pro‐fibrotic biomarkers such as αSMA.^56^, ^57^ TGF‐β overexpression in the aqueous humor leads to fibrosis of the trabecular meshwork, the outflow pathway of the eye.^54^, ^58^, ^59^ Earlier studies in normotensive rabbits found that blocking the lumen of GDIs to prevent aqueous outflow into the subconjunctival space reduces fibrosis.^23^ Thus, the Nano GDIs described in our studies may prevent fibrosis through both the nano‐architecture and degradable inner core that restricts outflow in the early post‐operative period. We found that nanofibers successfully induce resistance to TGF‐β‐ and LPA‐driven activation of human scleral fibroblasts in vitro, suggesting the response was regulated by a central signaling mechanism rather than receptor‐driven. Further, transcriptomic differences across actin‐regulated signaling, rho‐kinase signaling, and cytoskeletal regulation in fibroblasts cultured on nanofibers further suggested cell quiescence. Interestingly, *ITGA2*, a known collagen‐binding integrin highly expressed in tissue‐resident dendritic cells and macrophages,^18^, ^25^, ^60^ was significantly upregulated in fibroblasts cultured on nanofibers. The absence of *ITGA2* not only results in loss of the quiescence phenotype, but also activates cellular programs associated with cell migration in animal models of prostate, gastric, colorectal, and breast cancer.^61^, ^62^, ^63^ Indeed, in *ITGA2* knockout mice, ECM deposition was exacerbated in preclinical models of kidney fibrosis.^60^

Previously, Yu‐Wai‐Man et al. validated a cassette of transcripts that correlate with post‐operative fibrosis in the conjunctiva after glaucoma surgeries.^27^ In this genome‐wide RNA sequencing study analyzing patient‐derived fibroblasts, increased *MMP‐10*, *CD34*, and *IL‐33* expression and decreased *MYOCD* expression were most predictive of successful surgical outcomes.^27^ We observed that fibroblasts cultured on nanofibers potentiated a non‐fibrotic transcriptomic signature. Further, we observed that fibroblasts interacting with nanofibers displayed a reversible G0/G1 cell cycle arrest, and that extended nanofiber exposure resulted in fibroblasts exiting the cell cycle. Together, these findings suggest that biomaterials that mimic ECM could reduce fibroblast activation and promote more successful and durable surgical outcomes.

The in vivo performance of the Nano GDI in rabbits was favorable compared to existing surgical options. Due to the aggressive fibrotic response in rabbit eyes, normotensive rabbit models of glaucoma surgery are primarily used to evaluate biocompatibility, rate of fibrosis, and GDI functionality.^22^, ^64^ This likely explains why many implants are not rigorously evaluated for IOP reduction in preclinical studies prior to clinical evaluation. Clinically used GDIs comparable to the Nano GDI include the XEN Gel Stent, PRESERFLO™ MicroShunt, and STARflo Glaucoma Device (iSTAR Medical, Belgium). The XEN and PRESERFLO are small‐lumen tubes fabricated from porcine gelatin and poly(styrene‐block‐isobutylene‐block‐styrene), respectively. The material characteristics of these implants provide for stability and simple implantation. The XEN and PreserFlo reduced IOP by 30% and 25%, respectively, from baseline at 12 weeks in rabbit eyes when MMC was used, which is comparable to the performance of the nanofiber‐based GDI (9 mm Nano).^64^ The nanofiber‐based GDI has two features designed to optimize outcomes in the early and late post‐operative period: (1) the anti‐fibrotic nanofiber surface topography that promotes cell integration and reduces fibrotic signaling and (2) the expanding inner lumen which reduces outflow resistance as healing occurs. The STARflo implant is the only clinical‐stage implant with microstructural features that encourage cell infiltration.^65^ It is designed to vent aqueous humor into the suprachoroidal space through a porous microstructure. The microstructure of the STARflo is intended to encourage cell integration, creating a porous bed for fluid outflow. Without a central lumen, the STARflo relies on passive drainage of fluid through its pores which are at risk of occlusion by migrating cells. This strategy is dissimilar from the nanofiber‐based GDI, in which, as we demonstrated, the lumen undergoes gradual expansion, and the exterior wall is designed to reduce fibrosis by mimicking healthy ECM and integrating cells.

Incorporation of a nano‐architecture into GDIs is a promising strategy to impart advantageous physical cues and minimize biomaterials‐associated fibrosis to improve outcomes and extend functional lifespan without the need for secondary procedures or adjunct drug delivery systems. To construct Nano GDIs, we used PET and PU due to their favorable safety profile in humans.^66^, ^67^ PET and PU are hydrophobic, non‐degradable polymers which are resistant to protein deposition and esterase‐mediated hydrolysis, and have been used in engineering a wide range of biomedical implants from vascular grafts to sutures.^66^, ^67^ When PU GDIs were constructed with a nanofiber exterior, we observed that device migration into the eye was restricted as compared to smooth GDIs constructed from the same polymer. It is possible that in the acute post‐operative stage, the surface roughness of the nanofibers can provide an anchoring effect. In the later post‐operative stages, cellular integration may further prevent tube migration. Further, we found that nanofiber‐based GDIs retain their morphology and architecture for at least 1 year in vivo, and demonstrate significantly reduced fibrotic encapsulation with nanofiber‐based GDIs. Collectively, these results suggest that nanofiber‐based implants have potential to significantly improve glaucoma surgery outcomes, and may have application to other permanent implants.

## 4 CONCLUSIONS

Fibroblasts are a highly plastic cell type responsive to mechanical, chemical, and topographical cues. Myofibroblast transdifferentiation is associated with increased migration, proliferation, and ECM deposition that can cause complications following biomaterial implantation due to fibrotic encapsulation. ECM‐derived physical cues may have an important role in maintaining fibroblast quiescence. We showed nanofiber‐mediated attenuation of fibroblast activation and support of cellular quiescence. These cues mitigated induction of myofibroblast differentiation by pro‐fibrotic cytokines and induced a transcriptional signature predictive of surgical success. These promising in vitro findings were validated in a rabbit model of glaucoma surgery in which Nano GDIs achieved greater tissue integration, prevented hypotony, and mitigated subconjunctival fibrosis in comparison to smooth‐surfaced and commercially available GDIs. We propose that modulating fibroblast behavior through nanofiber coatings may reduce fibrotic encapsulation and increase functional GDI lifespan.

## 5 MATERIALS AND METHODS

### 5.1 Materials

Poly(ethylene terephthalate) (PET; inherent viscosity: 0.89 dl/g) was obtained from Nanofiber Solutions (OH, USA). Polyurethane (Chronoflex® C80 [medical device grade], PU) was obtained from Advansource Biomaterials (MA, USA). Polyglycolide (PGA [medical device grade]; inherent viscosity: 1.1 dl/g) was obtained from Corbion (NJ, USA). 1,1,1,3,3,3‐hexafluoro‐2‐propanol (HFIP), isopropyl alcohol, Triton X100, HEPES buffer, hexamethylenedisilazane (HMDS), fluorescein sodium, hydrochloric acid, bovine serum albumin (BSA) and xylene were purchased from Sigma Aldrich (MO, USA). Six‐well cell culture plates, phosphate buffered saline (PBS) Roswell Park Memorial Institute (RPMI) medium, fetal bovine serum (FBS), 4% paraformaldehyde (PFA), Dulbecco's modified eagle's medium (DMEM), penicillin/streptomycin (pen‐strep), sodium pyruvate, Prolong antifade gold mounting solution with DAPI, SYTOX deep red hardsetting mounting solution, 2% sodium cacodylate buffer, Fx cycle PI/RNase staining solution, sodium dodecylsulfate (SDS), Pierce™ BCA assay kit and 35‐mm Petri dishes were obtained from Thermo Fisher Scientific (MA, USA). All other cell culture supplies were purchased from Sigma Aldrich (MO, USA) and used unmodified unless otherwise stated. Antibodies for immunofluorescence staining were obtained from Thermo Fisher Scientific. Clones and catalog numbers are listed in Table S1. Twenty, 25, and 30‐gauge needles were purchased from Nordson EFD (OH, USA). TGF‐β and lysophosphatidic acid (LPA) were purchased from R&D Biosystems (MN, USA). Forward and reverse primer pairs were purchased from IDT (IA, USA). qRT‐PCR focal adhesion gene array was purchased from Qiagen (MD, USA). mRNA extraction was performed using RNeasy kit purchased from Qiagen (MD, USA) and reverse transcribed using a high‐capacity cDNA reverse transcription kit purchased from Applied Biosystems (MA, USA). Balanced saline solution (BSS) was obtained from Alcon (MD, USA). Ketamine hydrochloride was purchased from Dechra (KS, USA) and proparacaine hydrochloride was purchased from Bausch & Lomb (FL, USA). Betadine solution was obtained from Alcon (TX, USA). Mitomycin C (MMC) was purchased from Biosynth‐Carbosynth (CA, USA). Sutures used in the surgical procedures were purchased from Ethicon (NJ, USA). Syringes were obtained from Becton Dickinson and Company (NJ, USA). XEN® Gel Stent (XEN) was obtained from Allergan (Dublin, Ireland) and Baerveldt® Glaucoma Implant (BGI tube) tube material was obtained from Johnson & Johnson Vision (CA, USA). The template wires for GDIs and drill chuck and motor used in manufacturing GDIs were purchased from McMaster Carr (IL, USA).

### 5.2 Animal ethics statement

All animals were cared for and experiments conducted in accordance with protocols approved by the Animal Care and Use Committee of the Johns Hopkins University, the ARVO Statement for the Use of Animals in Ophthalmic and Vision Research, and the NIH Guide for the Care and Use of Laboratory Animals.

### 5.3 Scaffold manufacturing for in vitro experiments

PET was dissolved in HFIP at 10% (w/v) by stirring overnight at 50°C. PU was dissolved in HFIP at 6% (w/v). Following dissolution, the polymer solution was loaded into a 3 ml syringe and dispensed at a flow rate of 850 μl/h through a 20‐gauge stainless steel nozzle using a syringe pump. A voltage differential of 12.5 kV was maintained between the nozzle and a static aluminum collector. Nanofibers were cut into circular disks with a diameter of 3.5 cm (Figure S1a). Next, PET nanofibers were annealed at 120°C for 16 h and sterilized using isopropyl alcohol followed by UV exposure for 2 h. Smooth PET films were cut to 3.5 cm disks and sterilized using identical methods. Scaffolds were affixed to wells in a six‐well tissue culture plate prior to seeding. PU scaffolds were either annealed at 80°C in the nanofiber group or 150°C in smooth surface group. PU scaffolds underwent identical sterilization procedures to PET scaffolds prior to cell seeding. For protein adsorption, scaffolds were incubated with 1640 RPMI medium containing 10% FBS for 16 h. Following this, scaffolds were washed twice in PBS and adsorbed protein was extracted using 2% SDS. Protein concentrations were measured using BCA assay kit following the manufacturer's instructions.

### 5.4 Primary cell culture and maintenance

Primary human scleral fibroblasts were isolated and cultured as previously described.^4^ Briefly, eyes from non‐glaucomatous donors were received from the National Disease Research Interchange, dissected, and 1 × 1 mm scleral segments were placed in complete 1640 RPMI media supplemented with 10% FBS, non‐essential amino acids, 1% pen‐strep, and sodium pyruvate, inside collagen‐coated 35 mm Petri dishes for 14 days. Following this, cells were passaged and maintained in DMEM supplemented with 1% FBS, 1% pen‐strep, and sodium pyruvate. 1.5 × 10^5^ cells were seeded onto each scaffold and allowed to acclimatize for a period of 24 h prior to stimulation. Cells were used between passage 3 and 8 for all experiments.

### 5.5 Immunostaining cells

Forty‐eight hours after seeding, unstimulated cells were washed with sterile PBS, fixed in 4% PFA for 8 min, washed with sterile PBS, permeabilized using 0.1% Triton X100 for 15 min, and washed with sterile PBS again. After blocking with 3% BSA, cells were stained using phalloidin for 30 min, washed with 0.05% Tween 20 in PBS (PBST), and incubated with αTubulin primary antibody overnight at 4°C. After washing 3× with PBST, cells were incubated with Alexa Fluor 555 labeled secondary antibody for 2 h at room temperature and washed again 3× with PBST. Scaffolds were mounted onto glass slides and counterstained using prolong antifade mounting media containing either DAPI or Sytox red. Two‐dimensional (2D) images were obtained using a LSM 710 confocal laser scanning microscope (Zeiss, CA, USA) and processed using the Zen software (Blue, version 3.4). Z‐stacks were 3D reconstructed using IMARIS (Oxford Instruments, UK) to generate false volume images.

### 5.6 Scanning electron microscopy

Forty‐eight hours after seeding, unstimulated cells were fixed overnight at 4°C in buffered glutaraldehyde (2% [v/v] in sodium cacodylate buffer). Thereafter, cells were washed three times (5 min each) in HEPES buffer and subjected to an ethanol gradient (50%, 70%, 95%, and 100% ethanol, washed three times for 5 min in each solution). Following this, cells were chemically dried by HMDS exposure overnight. Samples were sputter coated with a 150 Å thick gold–palladium coating (Pd/Au). For imaging GDIs and blank scaffolds, samples were dried overnight under rough vacuum and subsequently sputter coated with Pd/Au. All samples were imaged using a Helios G4 UC Focused Ion Dual Beam scanning electron microscope (Thermo Scientific, MA, USA).

### 5.7 Atomic force microscopy

For roughness characterization, samples were manufactured as described above and adhered to glass coverslips. Samples were imaged using an MFP‐3D‐BIO Atomic Force Microscope (Asylum Research, Santa Barbara, CA), which was operated in AM (“tapping”) mode. At least three 40‐ or 90‐μm square regions were imaged for each sample. Fiji software (Life‐Line version 1.53c plus), with the “Roughness Calculation” plugin was used to calculate the arithmetic average roughness (Ra) values, and Gwyddion software (version 2.60) was used to generate 3D surface maps of the samples.

### 5.8 Mechanical testing

Rectangular cross‐sections of PU nanofiber and smooth scaffolds with a thickness of approximately 150 μm were cut in accordance with ASTM D882 testing standards. Samples were mounted onto an Instron tensile tester (model 5960) and pulled to the breaking point. Stress–strain curves were generated using the Bluehill3 (version 3.66) software. Young's modulus was estimated by measuring the slope of the elastic deformation regions using MATLAB (version 8.2.0.29).

### 5.9 Quantitative real‐time polymerase chain reaction analysis

Fibroblasts seeded on smooth and nanofiber scaffolds for 24 h were stimulated with either TGF‐β (2 ng/ml) or LPA (10 μM) for 24 h. RNA was extracted from cells using RNeasy Mini Kit using the manufacturer's instructions. mRNA levels were quantified using a spectrophotometer (NanoDrop, Thermo Scientific, MA, USA) and reverse transcribed using the high‐capacity cDNA reverse transcription kit according to the manufacturer's protocol. Ten ng of cDNA was analyzed per sample along with primer sequences listed in Table S2. For analyzing transcript levels associated with focal adhesion pathways, a RT2 PCR array was used. For analyzing transcripts from rabbit eye tissue, the sclera and conjunctiva of the bleb from each eye were dissected and weighed in separate tubes. Approximately 30 mg of tissue from each tube was pulverized and immediately placed in lysis buffer. RNeasy Mini Kit was used to obtain RNA from tissue using manufacturer's instructions. Fifty ng of cDNA was analyzed per sample along with primer sequences listed in Table S3. To perform all qRT‐PCR analysis, the ΔΔCt method was used. Expression was normalized to levels detected in untreated fibroblasts on smooth surfaces for in vitro studies and healthy, non‐operated conjunctiva, and sclera tissue in rabbit studies.

### 5.10 Cell cycle analysis

Fibroblasts were cultured in DMEM supplemented with 1% FBS, 1% pen‐strep, and sodium pyruvate for 24 h to synchronize cell cycles. Cells were then trypsinized and seeded on smooth (*n* = 9) and nanofiber scaffolds (*n* = 9) using a density of 150,000 cells per scaffold in six well plates. Twenty‐four hours after seeding, cells were washed twice in PBS, trypsinized and pooled into nanofiber and smooth scaffold groups. Cells were washed in PBS and ice‐cold methanol was added in drop‐wise fashion following which cells were maintained at −20°C for 15 min. Methanol was removed by centrifugation at 300*g* for 5 min followed by washing in PBS with 1% BSA. Finally, cells were stained with propidium iodide using FXcycle PI/RNAse staining solution for 30 min. Flow cytometry was performed on a Sony SH800 Cell Sorter (Sony, WA, USA) machine. Data were analyzed using FlowJo (FlowJo, version 10.8.1, LLC, OR, USA). Debris was gated out and singlet discrimination was performed before applying gating for propidium iodide positive cells. Cell cycle phases were annotated using the Cell Cycle plugin.

### 5.11 PET GDI manufacturing

PET GDIs were manufactured as described previously.^21^ Briefly, PGA solution (10% [w/v] in HFIP) was dispensed at a flow rate of 850 μl/h through a 20‐gauge nozzle. A potential difference of 12.5 kV was maintained between the nozzle and a template wire (outer diameter: 75 μm) affixed to a rotating drill chuck around which nanofibers were twisted to form a tubular structure with a thickness of 25 μm. This was to give the GDIs an initial inner diameter of 75 μm. Following this, PET nanofibers were deposited around the PGA layer under the same conditions until a final outer diameter of either 350 μm or 450 μm was attained and subsequently annealed at 100°C overnight.

### 5.12 PU GDI manufacturing

PGA nanofibers were deposited around a copper template wire, as described above, to form a degradable core. PU solutions (6% [w/v] in HFIP) were then dispensed at a flow rate of 850 μl/h through a 20‐gauge nozzle and a potential difference of 12 kV was maintained between the nozzle and the template wire (outer diameter: 75 μm). Following this PU nanofibers were deposited around the PGA core. Nanofibers were heated to 150°C to melt the PU nanofibers to form a smooth (thickness: 400 μm for smooth GDIs and 300 μm for Nanofiber GDIs), tubular structure. For GDIs with a nanofiber exterior, PU nanofibers were then electrospun around smooth PU GDIs. All GDIs were sterilized by exposure to isopropyl alcohol followed by UV irradiation for 2 h. A list of all GDIs along with dimensions and composition is in Table 1.

**TABLE 1:** Composition and dimensions of glaucoma drainage implants implanted in New Zealand White rabbit eyes

Shunt type	Polymer	Inner diameter (μm)	Outer diameter (μm)	Length (mm)
PCS1	Polyethyleneterephthalate	75–100	450	6
PCS2	Polyethyleneterephthalate	75–100	350	9
Smooth	Polyurethane	75–100	400	9
Nano	Polyurethane	75–100	400	9
Nano	Polyurethane	75–100	400	12
Baerveldt	Silicone	350	635	6
XEN	Porcine collagen	45	150	8

### 5.13 GDI implantation in normotensive rabbits

Healthy male and female New Zealand White rabbits (Robinson Services, NC, USA) were sedated by subcutaneous injection of ketamine:xylazine (75:5 mg/kg). A drop of 0.5% proparacaine hydrochloride ophthalmic solution followed by a drop of 5% betadine solution was administered to the operative eye. The eye was then draped in a sterile fashion. MMC was injected subconjunctivally (0.4 mg/ml; 100 μl) at the intended site of GDI placement. After placement of an 8‐0 silk, stay suture in the superotemporal limbus, a two‐clock hour, fornix‐based peritomy was created, and conjunctiva was dissected posteriorly. A 25 G needle was used to create a 2 mm scleral tunnel prior to entering the eye in the mid‐anterior chamber. The implant was gently guided through this tunnel. Once the GDI was in position, the template wire was removed, the external portion of the GDI was guided into the subconjunctival pocket, and a 10‐0 nylon cross‐stitch was placed at the site of tube entry to prevent peritubular leakage of aqueous humor. BSS was irrigated in the anterior chamber using a 30 G needle to validate implant patency. The conjunctiva was approximated to the limbus using two, 10‐0 nylon sutures and, again, BSS was irrigated into the anterior chamber to ensure that a water‐tight bleb formed. The distance from the internal tip of the implant within the anterior chamber to the corneal limbus was then measured. An identical procedure was performed to implant the BGI tube (6 mm length) and the XEN, with the exceptions that the scleral tunnels were created by a 23 G needle or the XEN Gel Stent handpiece, respectively. The stay suture was then removed and topical antibiotic ointment was administered to the eye. Surgeries were not performed in a masked manner due to the observable differences in appearance of the implants.

### 5.14 Rabbit IOP measurement and clinical evaluation

Successful outcomes of surgery were determined based on IOP, prevention of hypotony (IOP < 6 mmHg), thickness of the fibrotic capsule at POD 28, and expression of fibrosis‐related genes at POD 28. IOP was measured at baseline, 7, 14, 21, and 28 days after implantation, as previously reported.^21^ Briefly, IOP was measured using a TonoVet (iCare, Vantaa, Finland) rebound tonometer in awake, restrained rabbits without topical anesthesia between 10 a.m. and 12 p.m. by the same masked technician in the rabbit housing room. Each eye was measured three times. If measurements differed by >2 mmHg, the rabbit was allowed to acclimatize for three additional minutes prior to repeating the measurement. For all experiments, hypotony was defined as IOP <6 mmHg and a clinical presentation of a shallow anterior chamber. AUC_last_ values for IOP lowering were calculated using the software Prism (GraphPad, version 9). Fourteen and 28 days after implantation, bleb morphology was graded by examining sedated animals using the Indiana Bleb Appearance Grading Scale (IBAGS) to assign numerical scores for height, extent, and vascularity based on standardized photos. This method has previously been used in rabbit models of glaucoma surgery to evaluate bleb morphology in response to small molecule therapies and device‐specific modifications in preventing scar formation.^68^, ^69^, ^70^, ^71^ Implant migration was assessed by measuring the distance between the distal end of the GDI within the anterior chamber to the corneal limbus at baseline immediately after implantation, and then at 14 and 28 days post implantation. For assessing GDI patency, rabbits were sedated and proparacaine hydrochloride drops were administered topically. Afterwards, 100 μl of 0.01% (w/v) fluorescein sodium was injected into the anterior chamber. Black light illumination was used to photograph whether the fluorescein flowed out of the anterior chamber through a patent GDI.

### 5.15 Histology and immunostaining tissue

At the endpoint of the animal studies, rabbits were euthanized and eyes were enucleated. Conjunctival and scleral issue surrounding the implant was dissected and stored in formalin until further processing. Tissues were embedded in paraffin and allowed to cool. Five μm sections of tissues in paraffin blocks were mounted on microscope slides. Hemotoxylin and eosin (H&E) as well as Masson's trichrome (MT) staining was performed using standard protocols. For immunostaining, H&E‐stained sections were de‐stained by exposure to 1% (v/v) hydrochloric acid in ethanol for approximately 1 min. Tissue was deparaffinized in xylene and washed with 1:1 solution of ethanol in xylene. Subsequently, tissue sections were exposed to an ethanol gradient (100%, 90%, 80%, 70%, 50% ethanol in water) followed by rinsing gently in deionized water. Following this, antigen retrieval was performed using a trypsin‐based antigen retrieval kit (Abcam, Cambridge, UK) following the manufacturer's protocol. Following antigen retrieval, tissue was stained overnight with a mouse monoclonal alpha‐smooth muscle actin (αSMA) (1A4 [asm‐1]) primary antibody at 4°C. After washing three times for 5 min each in PBST, tissue was incubated at room temperature with a secondary antibody conjugated to Alexa Fluor 555 for 2 h. Finally, samples were counterstained with SYTOX red and mounted on glass slides using an antifade mounting solution.

### 5.16 Image analysis

H&E and MT images were obtained using a NIKON (Eclipse Ni) light microscope and NIS elements imaging software (Nikon, IL, USA, version 5.11.0). RGB histograms were generated using ImageJ (version 1.53k14) and collagen intensity was recorded from the blue channel. Immunofluorescence images were obtained using a confocal laser scanning microscope (Ziess, LSM710), processed using the Zen imaging software (Blue, version 3.4) and quantified using ImageJ. Image analysis and quantification were conducted in a masked manner using at least three images per animal and averaged.

### 5.17 Statistical methods

All statistical analyses were performed using GraphPad Prism (ver. 9.0). To compare data sets with two groups, a two‐tailed student's *t* test was used, and to compare data sets with multiple groups, analysis of variance (ANOVA) was used. Unless otherwise mentioned, data are represented as mean ± SD. All experiments were performed with at least three replicates. For qRT‐PCR analysis, three independent experiments were performed and averaged. For cell cycle analysis, histograms are from six independent samples that were pooled and analyzed. All collected data were included in the analyses.

## AUTHOR CONTRIBUTIONS

**Aditya Josyula:** Data curation (lead); methodology (lead); validation (lead); visualization (lead); writing – original draft (lead); writing – review and editing (lead). **Ann Mozzer:** Methodology (supporting); validation (supporting). **Julia Szeto:** Data curation (supporting); methodology (supporting); validation (supporting). **Youlim Ha:** Data curation (supporting); investigation (supporting); methodology (supporting). **Nicole Richmond:** Data curation (supporting); formal analysis (supporting); methodology (supporting). **Seung Woo Chung:** Data curation (supporting); formal analysis (supporting); methodology (supporting). **Sri Vishnu Kiran Rompicharla:** Data curation (supporting); investigation (supporting); methodology (supporting). **Janani Narayan:** Data curation (supporting); investigation (supporting); methodology (supporting). **Samiksha Ramesh:** Data curation (supporting); investigation (supporting); methodology (supporting). **Justin Hanes:** Conceptualization (supporting); funding acquisition (supporting); resources (supporting); supervision (supporting). **Laura Ensign:** Conceptualization (supporting); funding acquisition (supporting); investigation (supporting); resources (supporting); supervision (supporting); writing – original draft (supporting); writing – review and editing (supporting). **Kunal Parikh:** Conceptualization (lead); funding acquisition (supporting); investigation (equal); methodology (equal); project administration (lead); resources (supporting); supervision (equal); writing – original draft (supporting); writing – review and editing (supporting). **Ian Pitha:** Funding acquisition (lead); investigation (lead); resources (lead); supervision (lead); writing – original draft (supporting); writing – review and editing (supporting).

## CONFLICT OF INTEREST

Kunal Parikh, Ian Pitha, and Justin Hanes are inventors on pending US and international patents (US201662306848P, EP17715299.8A, JP2021126536A, PCT/US2017/022090) related to development and use of the technology. Terms of any future commercial arrangements will be managed by Johns Hopkins University which is the current assignee on the aforementioned patents. All other authors declare no competing interests.

### PEER REVIEW

The peer review history for this article is available at https://publons.com/publon/10.1002/btm2.10487.

## Supporting information


**Data S1.** Supporting Information.

## Data Availability

All data, code, and materials used in this study and analyses are available within the paper or supplementary materials.
