# Supplementary material for: Nanofiber‐based glaucoma drainage implant improves surgical outcomes by modulating fibroblast behavior
Source: Bioeng Transl Med. 2023 Jan 18;8(3):e10487. doi: 10.1002/btm2.10487 (PMC10189467; doi:10.1002/btm2.10487)
Supplement: Supplementary file 1 — Data S1. Supporting Information. [file BTM2-8-e10487-s001.docx]

**Supplementary Materials**

**List of Supplementary Materials:**

Fig. S1. Manufacturing and characterization of nanofiber and smooth scaffolds.

Fig. S2. Cell shape and size analysis.

Fig. S3. Transcriptomic profile of fibrosis associated genes in human scleral fibroblasts *in vitro.*

Fig. S4. Cell cycle analysis of fibroblasts cultured on nanofiber and smooth scaffolds for 48 hours.

Fig. S5. Comparative evaluation of smooth and nano GDIs *in vivo*.

Fig. S6. Comparative evaluation of Nano and clinically available GDIs *in vivo*.

Table S1. Antibodies and clones used for immunofluorescent staining.

Table S2. Forward and reverse primer sequences used for qRT-PCR analysis of primary human scleral fibroblasts.

Table S3. Forward and reverse primer sequences used for qRT-PCR analysis of rabbit conjunctival and scleral tissue.

Table S4. P-value table generated using one-way ANOVA analysis from qRT-PCR analysis of scleral and conjunctival tissue from blebs. Statistically significant differences are highlighted in blue.

**
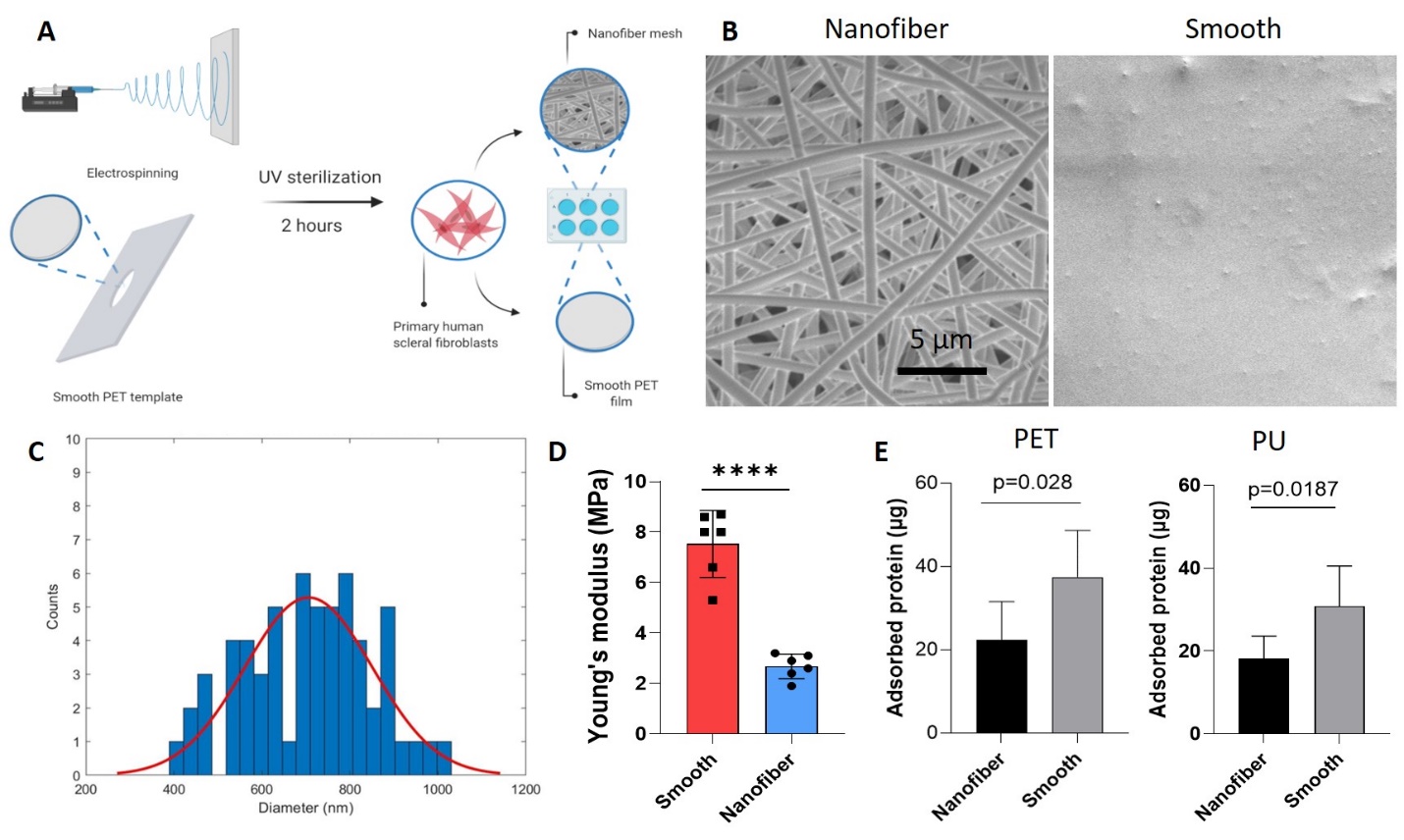
**

**Fig. S1. Manufacturing and characterization of nanofiber and smooth scaffolds.** (A) Schematic of scaffold manufacturing and cell studies. (B) SEM images of nanofiber and smooth PET scaffolds. (C) Size distribution of nanofibers in PET scaffolds. (D) Young’s modulus of scaffolds constructed using PET. (E) Protein adsorption on PET and PU scaffolds. P-value was calculated by student’s t-test. **p* ≤ 0.05, ***p* ≤ 0.01, ****p* ≤ 0.001, *****p* ≤ 0.0001 by student’s t-test for data in D and E.


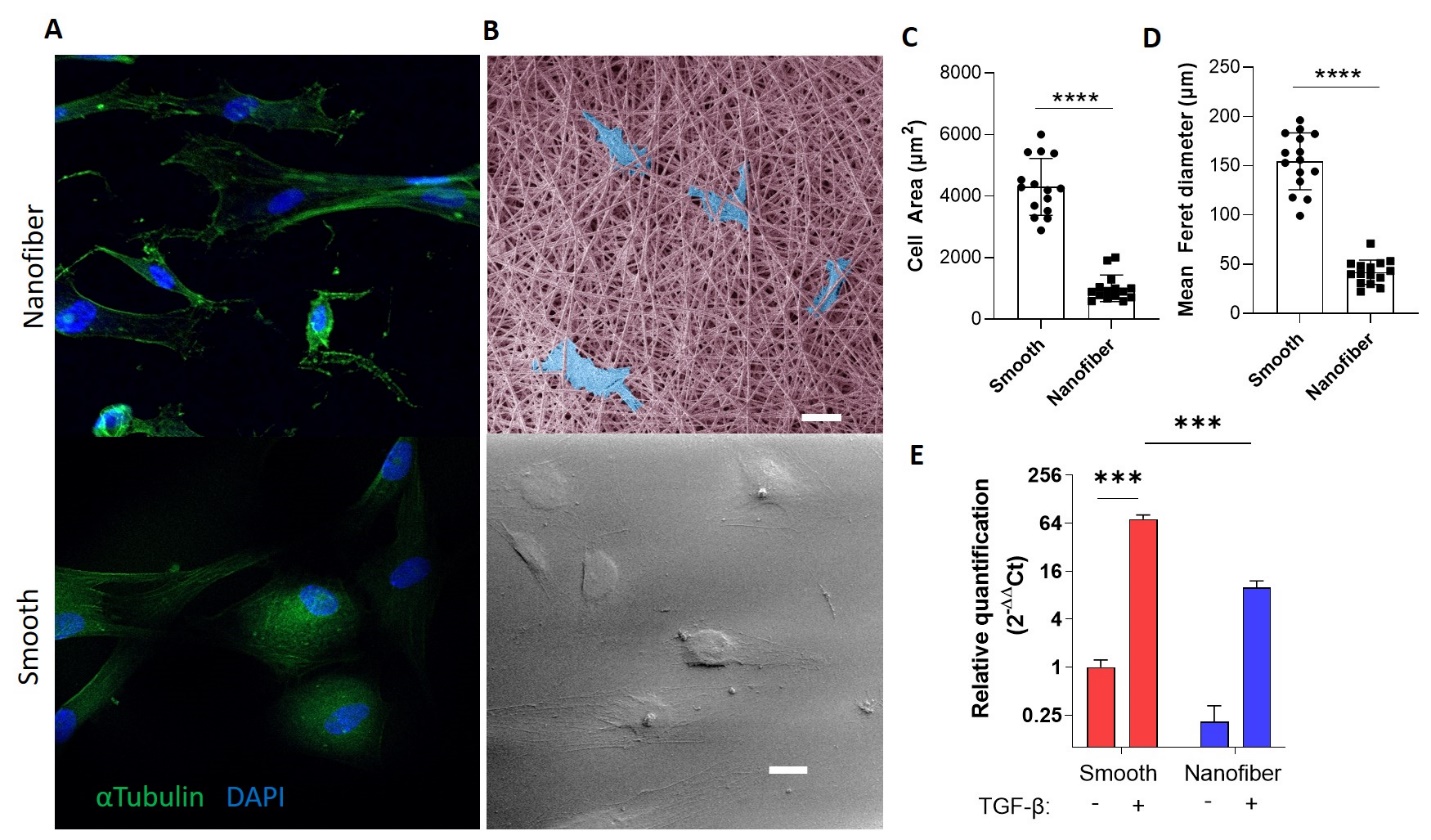


**Fig. S2.** **Cell shape and size analysis.** (A) Confocal microscopy of fibroblasts on nanofiber and smooth PU scaffolds. (B) SEM images showing human scleral fibroblasts on nanofiber and smooth scaffolds. (C) Cell area and (D) mean ferret diameter of fibroblasts on nanofiber scaffolds were significantly reduced compared to smooth scaffolds. Scale bars represent 10 μm. (E) αSMA expression of unstimulated and TGF-β stimulated fibroblasts cultured on PU scaffolds. **p* ≤ 0.05, ***p* ≤ 0.01, ****p* ≤ 0.001, *****p* ≤ 0.0001 by student’s t-test for data in C, D and E.


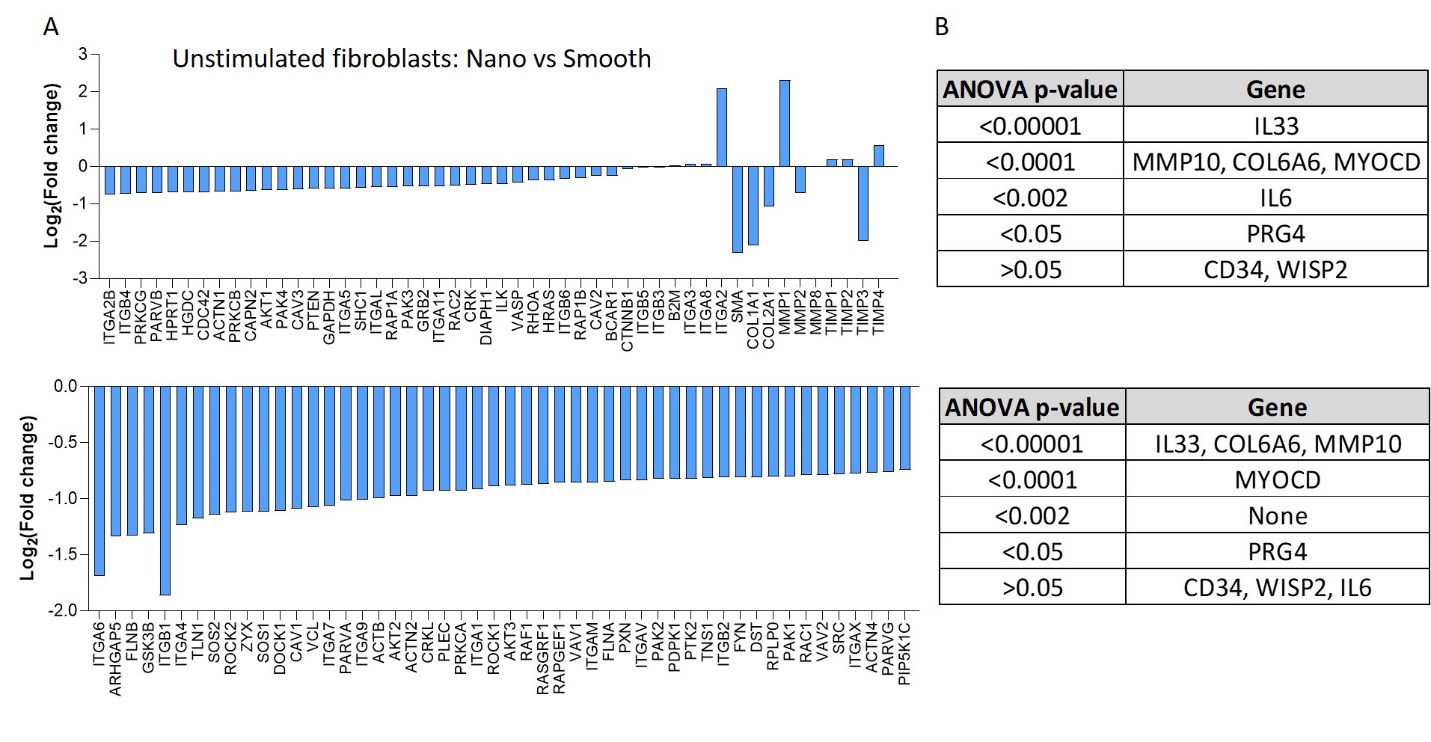


**Fig. S3. Transcriptomic profile of fibrosis associated genes in human scleral fibroblasts *in vitro*** (**A**) Differential expression of fibrosis associated genes in fibroblasts cultured on nanofiber scaffolds relative to fibroblasts cultured on smooth scaffolds. (**B**) ANOVA p-values of specific transcript expression levels for cells with and without TGF-β stimulation.


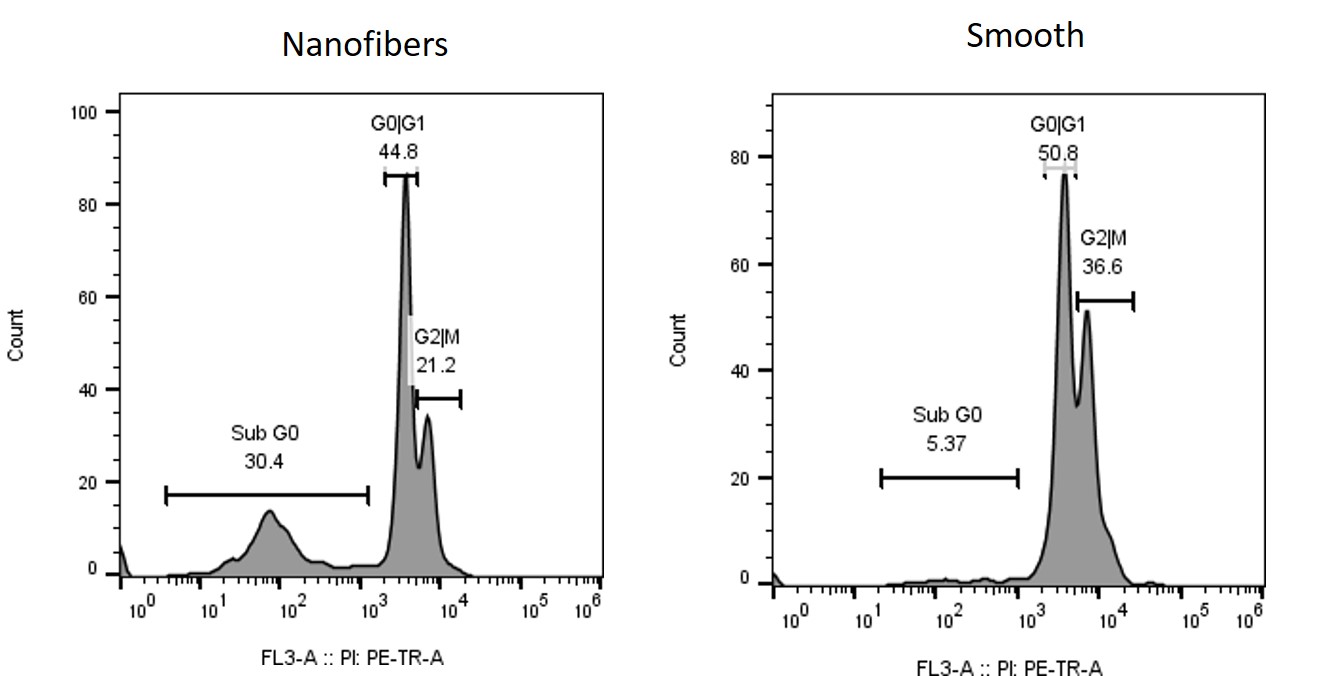


**Fig. S4.** Cell cycle analysis of fibroblasts cultured on nanofiber and smooth scaffolds for 48 hours.

**
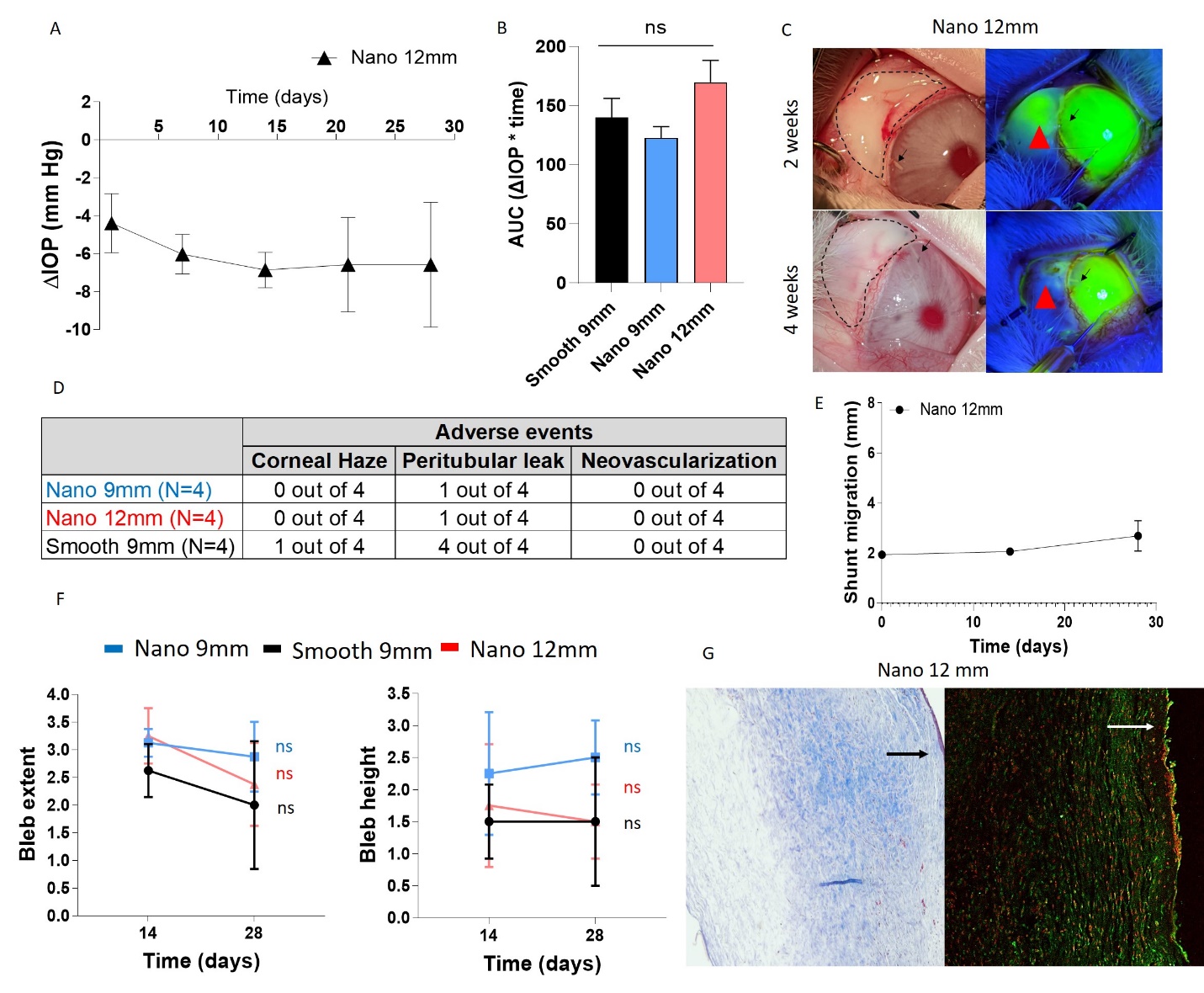
**

**Fig. S5. Comparative evaluation of smooth and nano GDIs *in vivo*.** (**A**) IOP lowering over 28 days in NZW rabbits achieved by 12 mm long Nano GDI. (**B**) Cumulative IOP reduction (AUC) over 28 days for smooth and nanofiber-based GDIs. (**C**) Gross images of subconjunctival blebs (left) and GDI patency assessment via anterior chamber irrigation with fluorescein sodium (right) at 2 weeks and 4 weeks post-operatively. (**D**) Table of adverse events observed in each group during the 28-day observation period. (**E**) 12 mm long Nano GDI migration over 28 day observation period. (**F**) Bleb morphology of GDIs at post-operative days 14 and 28. (**G**) Representative Masson’s trichrome (left) and immunofluorescence (right) images of tissue surrounding Nano GDIs (12mm length) at post-operative day 28.


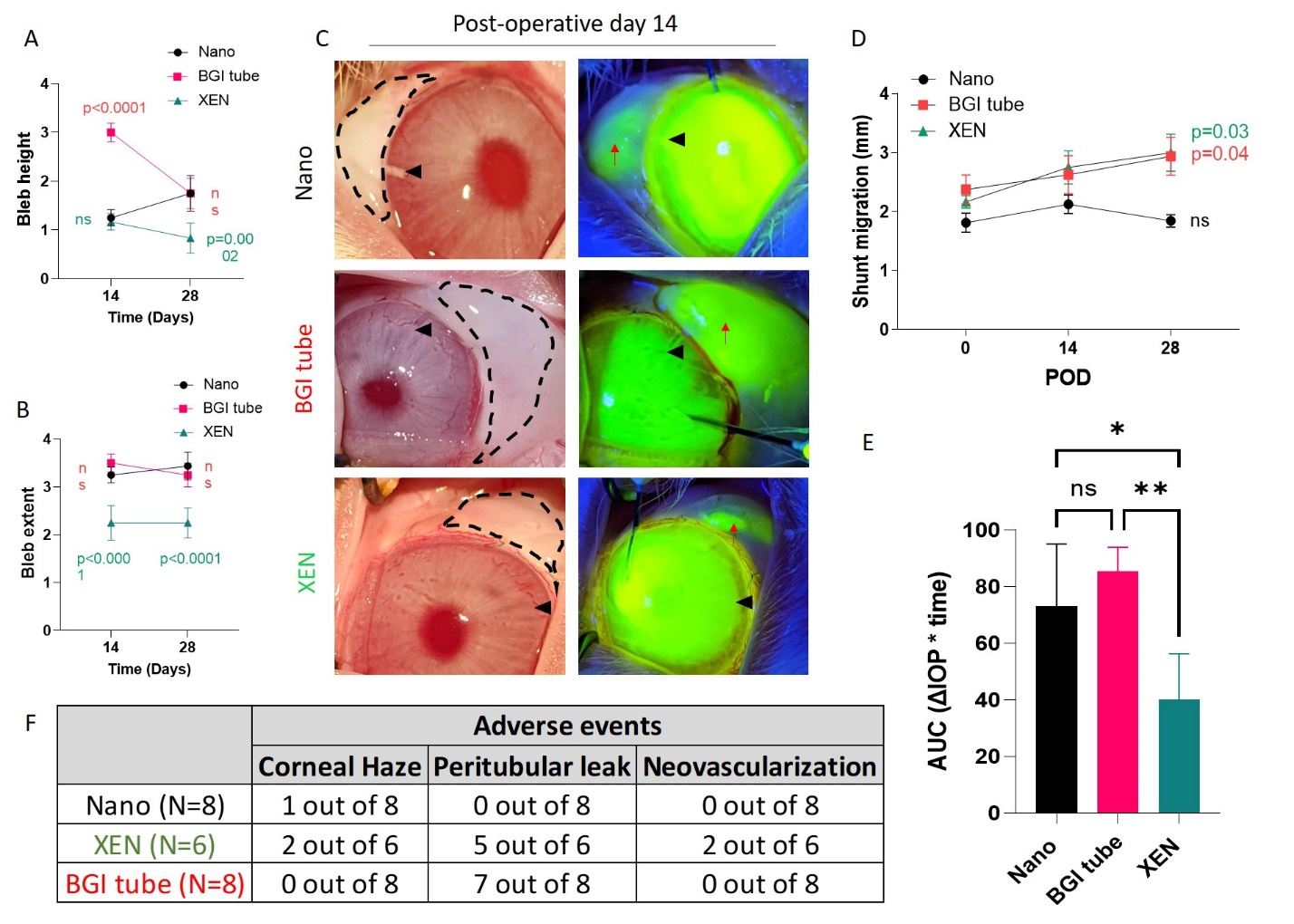


**Fig.** **S6. Comparative evaluation of Nano and clinically available GDIs *in vivo*.** (**A**) Bleb height and (**B**) extent measurements showed Nano and BGI tube groups had blebs with significantly greater elevation and extent as compared to XEN at post-operative day 28. (**C**) Active subconjunctival drainage as confirmed by anterior chamber irrigation with fluorescein sodium (right) and representative gross images of subconjunctival blebs (left) 28 days after GDI placement. Black arrowheads show distal end of GDIs in the anterior chamber and red arrows indicate subconjunctival drainage. (**D**) Cumulative IOP reduction (AUC) over 28 days. (**E**) Measurements of GDI migration at post-operative days 14 and 28. (**F**) Table describing adverse events observed during clinical evaluation of GDIs. P-values were calculated using ANOVA for data in **A, B** and **E** and two-tailed student’s T-test in **D.**

**Table S1.** Antibodies and clones used for immunofluorescent staining.

**Table S2.** Forward and reverse primer sequences used for qRT-PCR analysis of primary human scleral fibroblasts.

**Table S3.** Forward and reverse primer sequences used for qRT-PCR analysis of rabbit conjunctival and scleral tissue.

**Table S4.** P-value table generated using one-way ANOVA analysis from qRT-PCR analysis of scleral and conjunctival tissue from blebs. Statistically significant differences are highlighted in blue.
